# Correction: Itaconate and derivatives reduce interferon responses and inflammation in influenza A virus infection

**DOI:** 10.1371/journal.ppat.1011002

**Published:** 2022-11-29

**Authors:** Aaqib Sohail, Azeem A. Iqbal, Nishika Sahini, Fangfang Chen, Mohamed Tantawy, Syed F. H. Waqas, Moritz Winterhoff, Thomas Ebensen, Kristin Schultz, Robert Geffers, Klaus Schughart, Matthias Preusse, Mahmoud Shehata, Heike Bähre, Marina C. Pils, Carlos A. Guzman, Ahmed Mostafa, Stephan Pleschka, Christine Falk, Alessandro Michelucci, Frank Pessler

In Fig 6, the x-axis label for the IAV row in panel C is incorrect. The authors have provided a corrected version of Fig 6 here.

**Fig 6:**
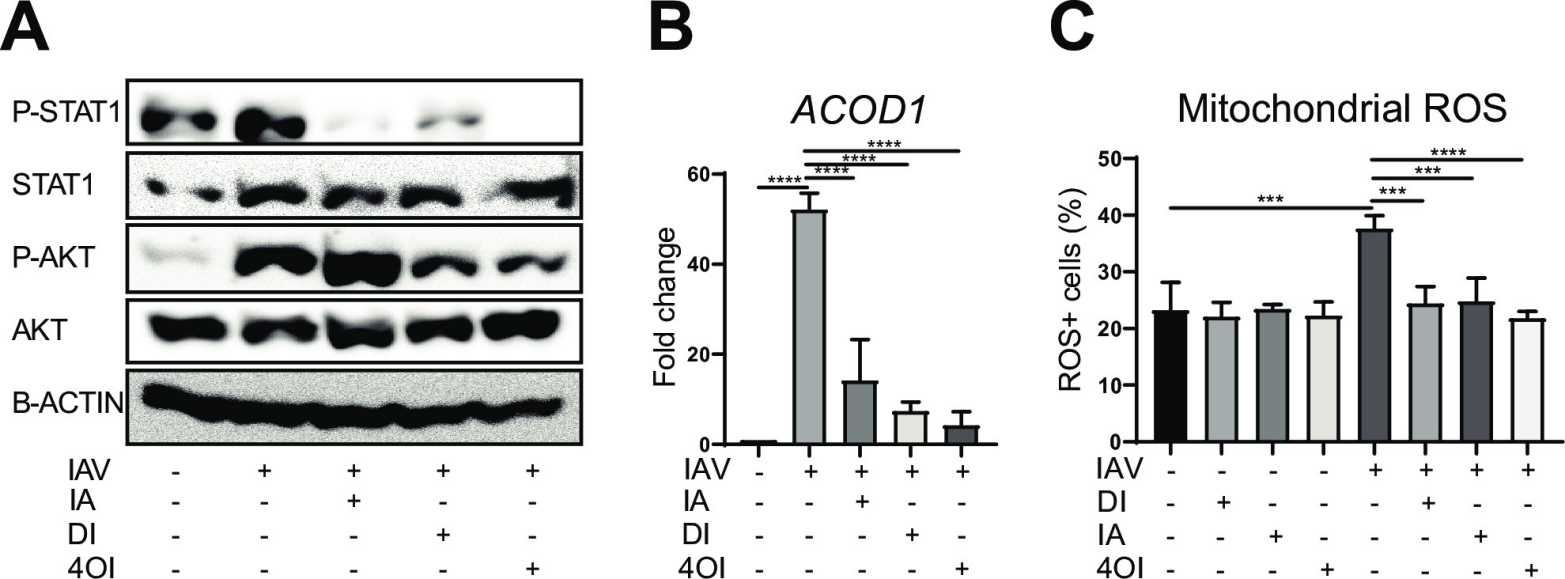
Effects of itaconates on phosphorylation of STAT1 and AKT, *ACOD1* mRNA expression, and ROS levels. Treatments (itaconate, 20 mM; DI 0.5 mM; 4OI 125 μM) were applied as indicated to dTHP1 cells infected with IAV (PR8M, MOI = 1), and analyses performed 12 h p.i. **A.** Itaconate, DI, and 4OI inhibit STAT1 phosphorylation, but only DI and 4OI inhibit AKT phosphorylation (immunoblot for the indicated targets, using β-actin as internal control). **B.** 4OI exerts the strongest *ACOD1* mRNA reduction (RT-qPCR, n = 3). Reference for fold change = uninfected cells 12 h. **C.** The three itaconates reduce IAV-induced mitochondrial ROS levels to a similar degree (flow cytometry, n = 3). Mean ±SEM *p<0.05; **p<0.01; ***p<0.001 (1-way ANOVA followed by Tukey’s multiple comparison test).
