# Supplementary figures and images for: Itaconate and derivatives reduce interferon responses and inflammation in influenza A virus infection
Source: PLoS Pathog. 2022 Jan 13;18(1):e1010219. doi: 10.1371/journal.ppat.1010219 (PMC8846506; doi:10.1371/journal.ppat.1010219)

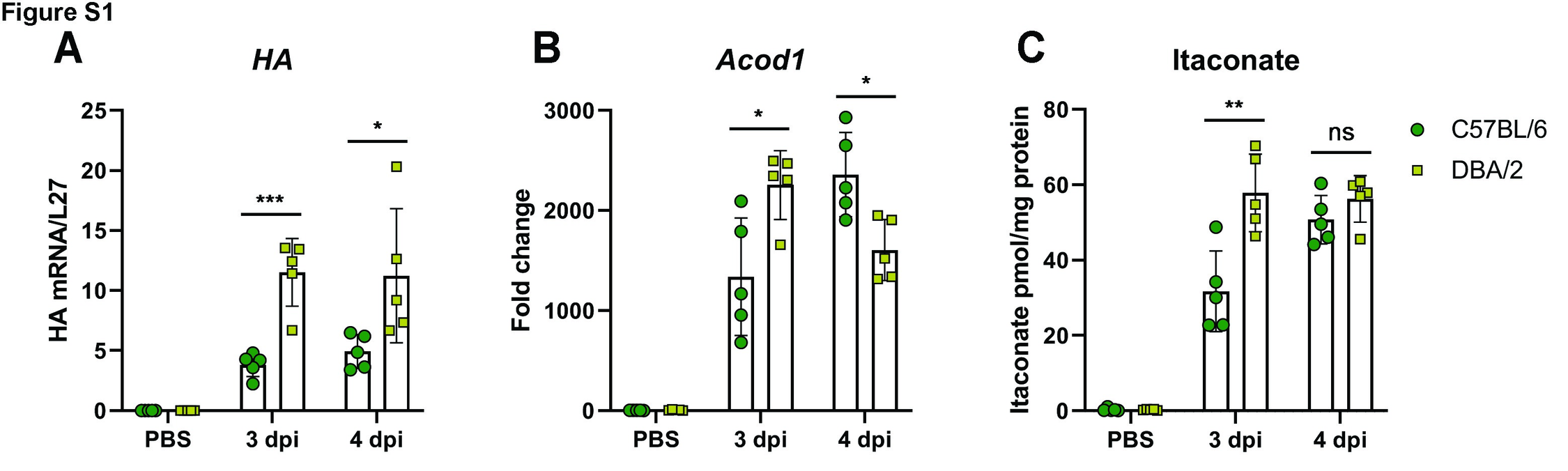

Supplement: S1 Fig — Mice were infected with IAV strain PR8M (2x10^3^ FFU) as described in (31). Levels of HA and Acod1 mRNA (RT-qPCR) and itaconate (LC-MS/MS) were measured on days 3 and 4 p.i. Lungs collected 3 days after mock infection with PBS were used as controls. Means ±SD. *p<0.05; **p<0.01 (t test). A. HA mRNA. B. Acod1 mRNA. Reference for fold change = PBS control of the respective mouse strain. C. Itaconate. (TIF) [file ppat.1010219.s001.tif]

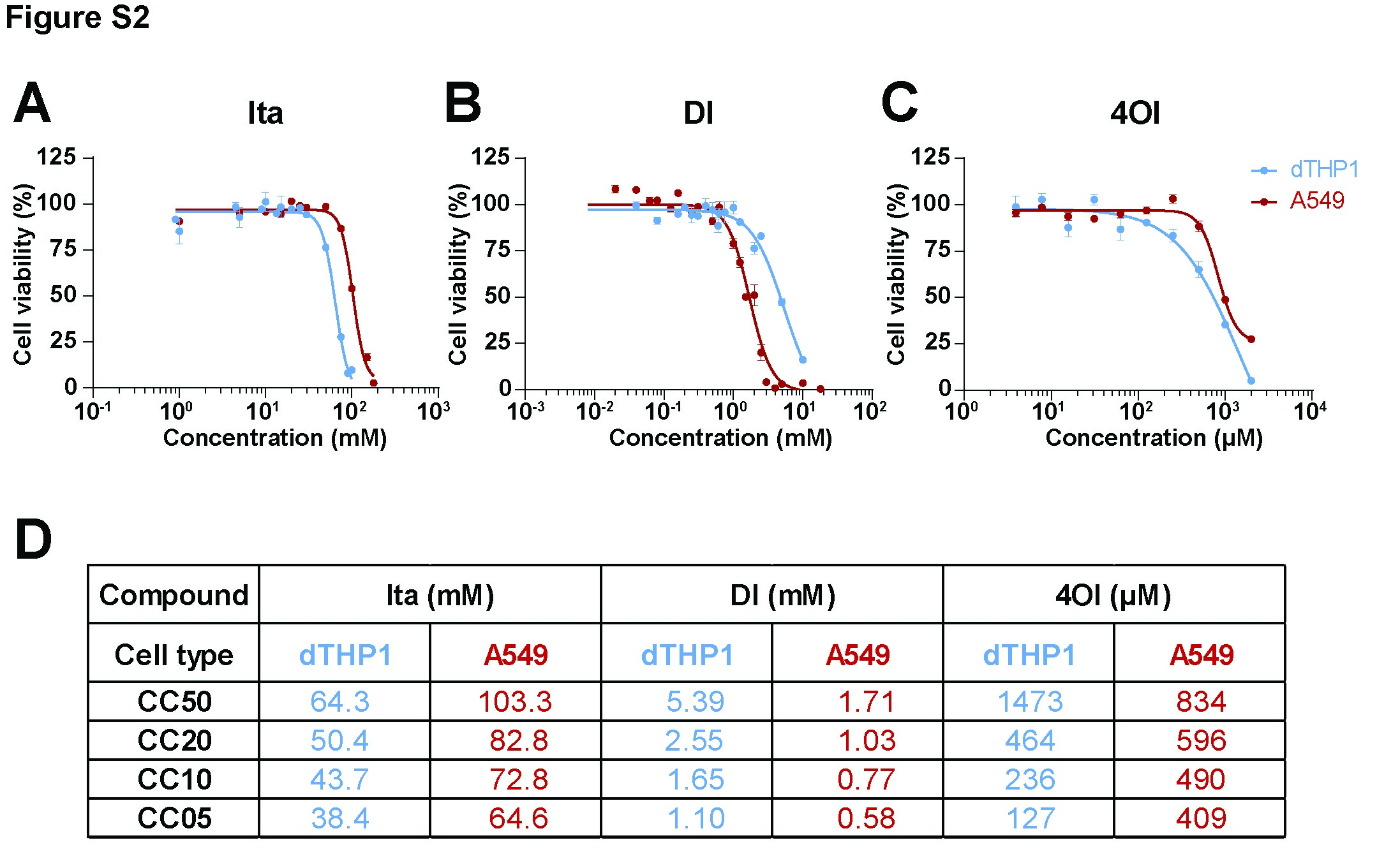

Supplement: S2 Fig — Cytotoxicity was determined by applying increasing concentrations of the compounds to dTHP1 (25 x103 cells/well) and A549 cells (20 x103 cells/well) and measuring cell respiration with the 3-(4,5-dimethylthiazol-2-yl)-2,5-diphenyltetrazolium bromide (MTT) assay after 24 h. Curves for dTHP1 and A549 cells are shown in the same graphs for comparison, illustrating that itaconate and 4OI tend to be more toxic on dTHP1 cells, whereas DI is more toxic on A549 cells. A. Itaconate (n = 3 experiments, each with n = 8 replicates). B. DI (n = 3 experiments, each with n = 8 replicates). C. 4OI (n = 1 experiment with n = 8 replicates). Means ±SEM. D. Table summarizing the concentrations at which mean viability was reduced by 5, 10, 20 or 50%. The concentrations used in this study were selected to be < CC05. CC = concentration at which a given percentage of cells was non-viable. (TIF) [file ppat.1010219.s002.tif]

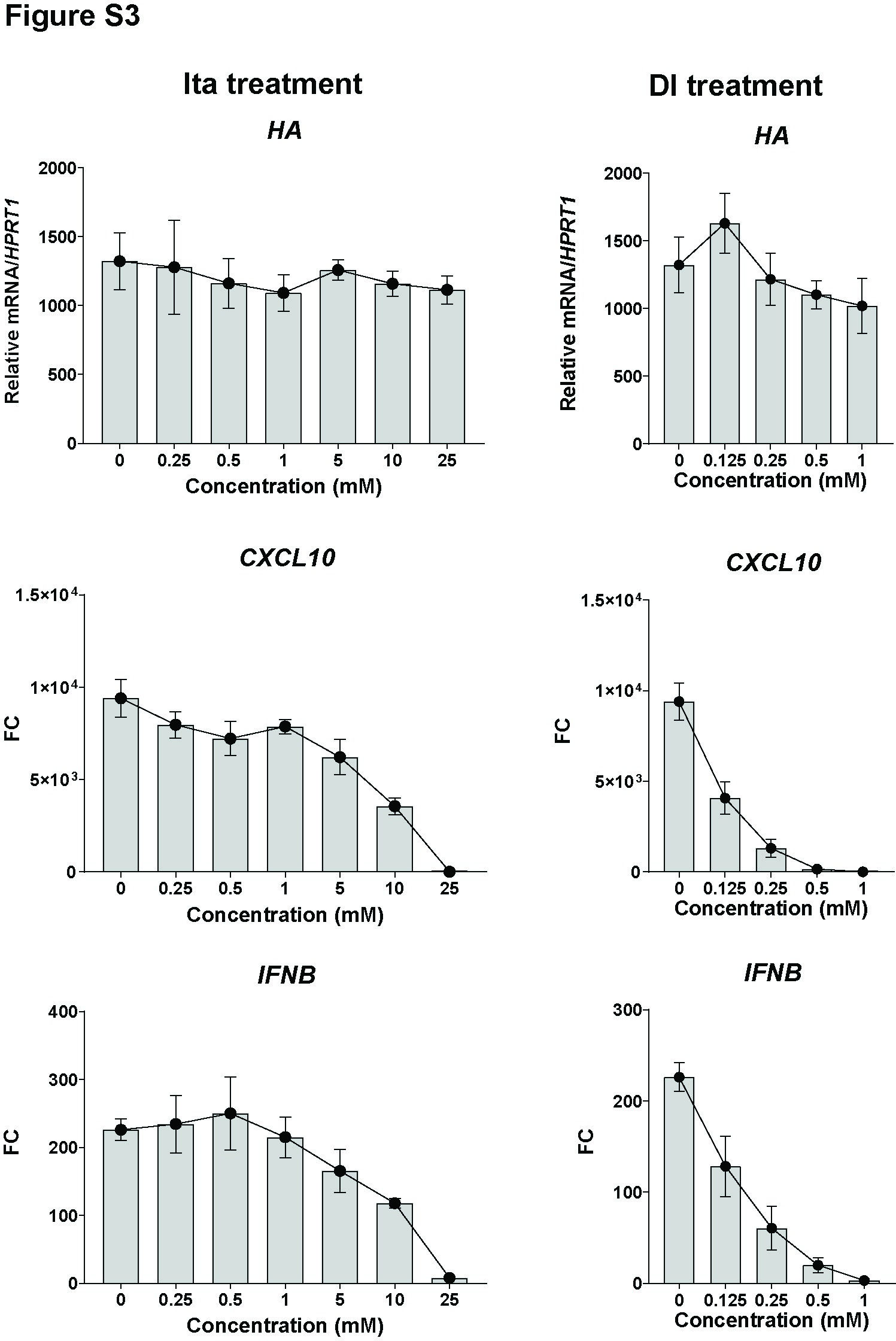

Supplement: S3 Fig — dTHP-1 cells were pretreated overnight with the indicated concentrations of itaconate or DI, infected with IAV (PR8M; MOI = 1), and expression of HA and CXCL10 mRNA was measured after 12 h (RT-qPCR) (n = 3). Reference for fold change = uninfected, untreated 12 h. (TIF) [file ppat.1010219.s003.tif]

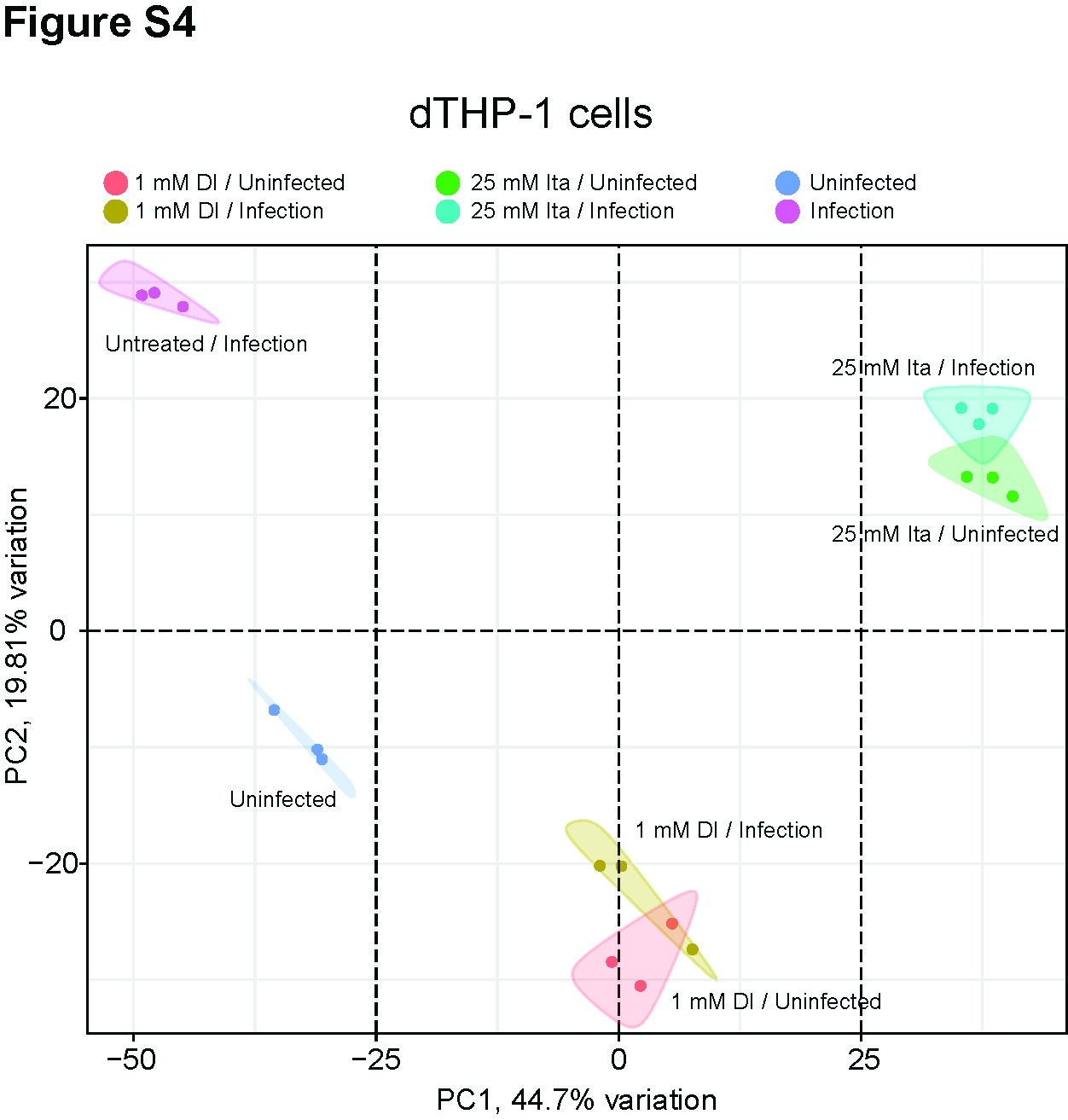

Supplement: S4 Fig — dTHP1 cells were infected with IAV (PR8M, MOI = 1) and gene expression assessed by microarray analysis 12 h p.i. PCA based on the microarray analysis shown in Fig 3. Untreated and treated IAV-infected cells cluster together, whereas untreated infected cells are clearly separated from all other groups in both principle components. (TIF) [file ppat.1010219.s004.tif]

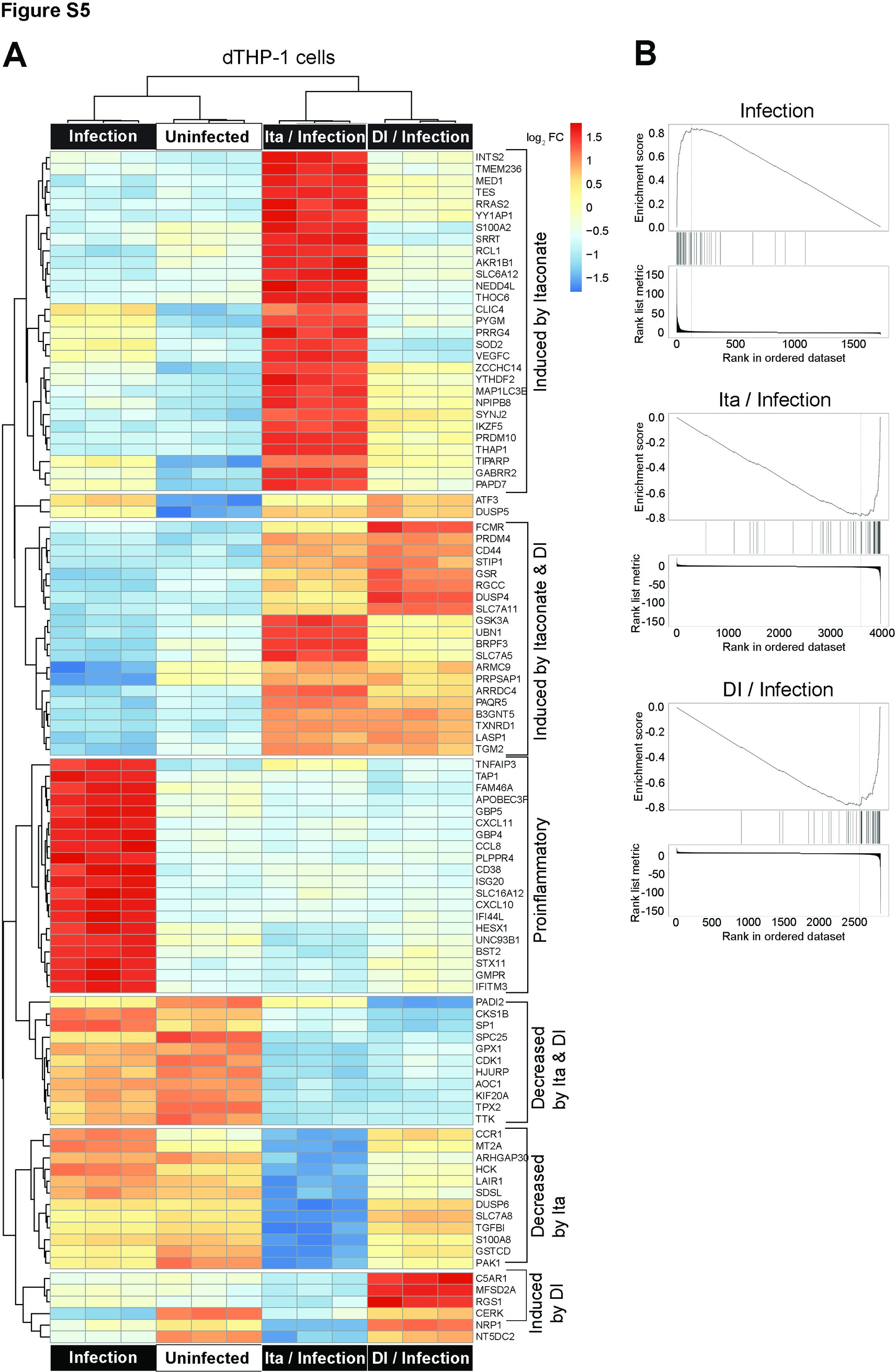

Supplement: S5 Fig — A. Hierarchical clustering analysis of effects of itaconate and DI on IAV-infected dTHP-1 cells. dTHP1 cells were infected with IAV (PR8M, MOI = 1) and gene expression assessed by microarray analysis 12 h p.i. Analysis based on the same microarray data as used for Figs 3 and S4, using the top 100 DEGs (FDR F-test <1.89E-09). Uninfected cells and untreated IAV-infected cells form one clade and itaconate and DI treated IAV-infected cells the other clade. From top to bottom the brackets identify the following clades: (i) a large clade of DEGs that are upregulated upon itaconate treatment, (ii) DEGs that are upregulated by both itaconate and DI, (iii) a predominantly pro-inflammatory clade that is induced in IAV infection and globally downregulated by both treatments, (iv) DEGs downregulated by both itaconate and DI, (v) DEGs downregulated only by itaconate, (vi) DEGs upregulated only by DI. B. Enrichment plots based on the 99 genes contained in GO term Response to type I interferon, based on the microarray data used for Figs 3, S4, and S5A. Enrichment scores are plotted on the y-axis, the mRNAs (identified by vertical lines) along the x-axis, ranked by fold change. The plots illustrate pronounced enrichment in a large number of genes due to infection, which is nearly quantitatively depleted by treatment with both itaconate and DI. (TIF) [file ppat.1010219.s005.tif]

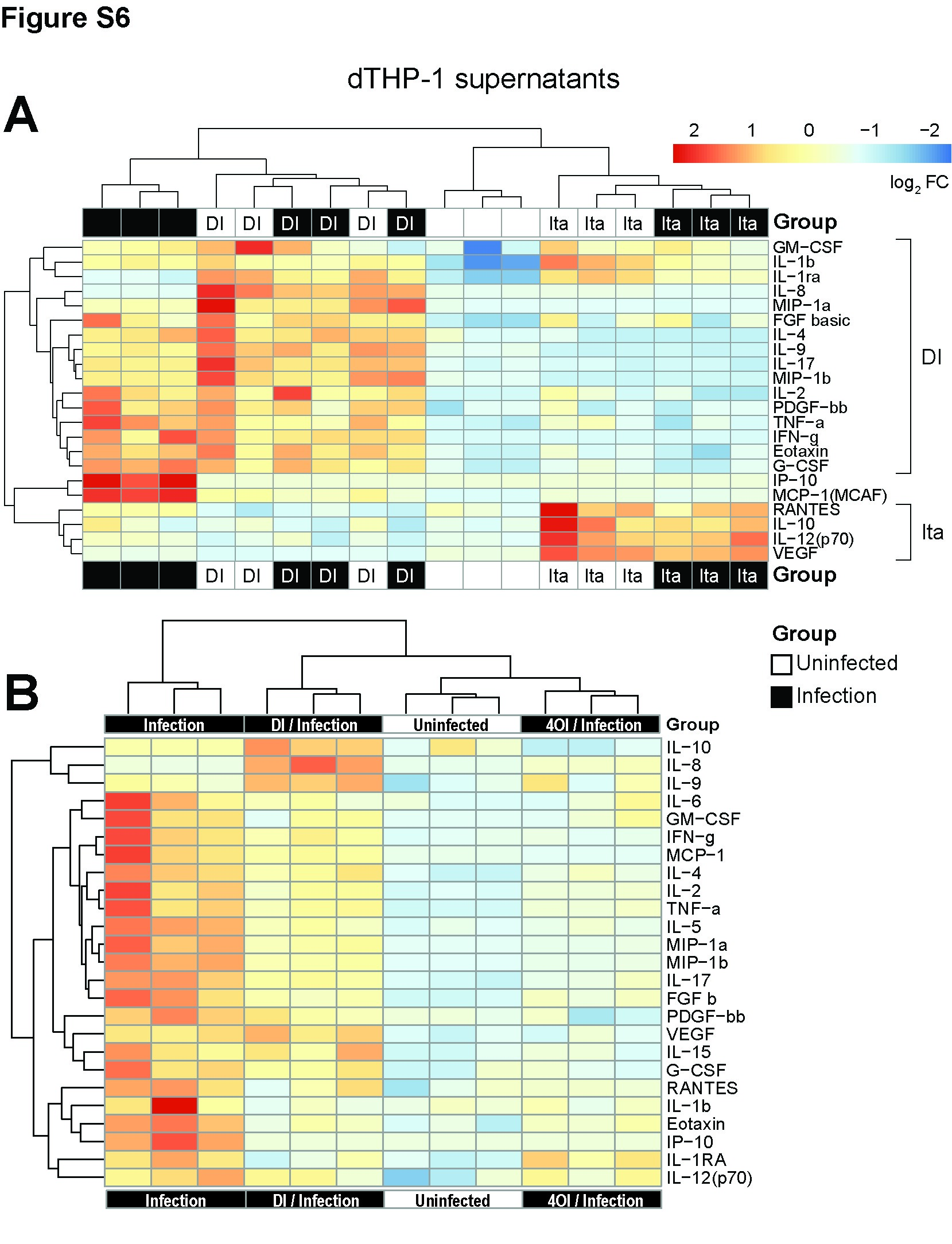

Supplement: S6 Fig — A. Supernatants of uninfected and IAV-infected (PR8M, MOI = 1) dTHP1 cells, with or without itaconate or DI treatment, were analyzed for concentrations of 27 cytokines/chemokines by multiplex microbead array 12 h after infection or mock treatment. A hierarchical clustering analysis was carried out with those targets that were detected above limit of detection of the assay in most samples. The microarray analysis of cellular gene expression in the same experiment is shown in Fig 3 (n = 3). B. Supernatants of uninfected and IAV-infected (PR8M, MOI = 1) dTHP1 cells, with our without DI or 4OI treatment, were analyzed 12 h p.i. with the same 27-plex assay as in A. More targets were detected > LOD than in A, the effect of DI was, overall somewhat more pronounced, and DI treatment increased IL-10 levels in addition to IL-8 and IL-9. 4OI differs from DI in that it reduces levels of most targets more efficiently and does not increase IL-8, -9, and -10 levels. (TIF) [file ppat.1010219.s006.tif]

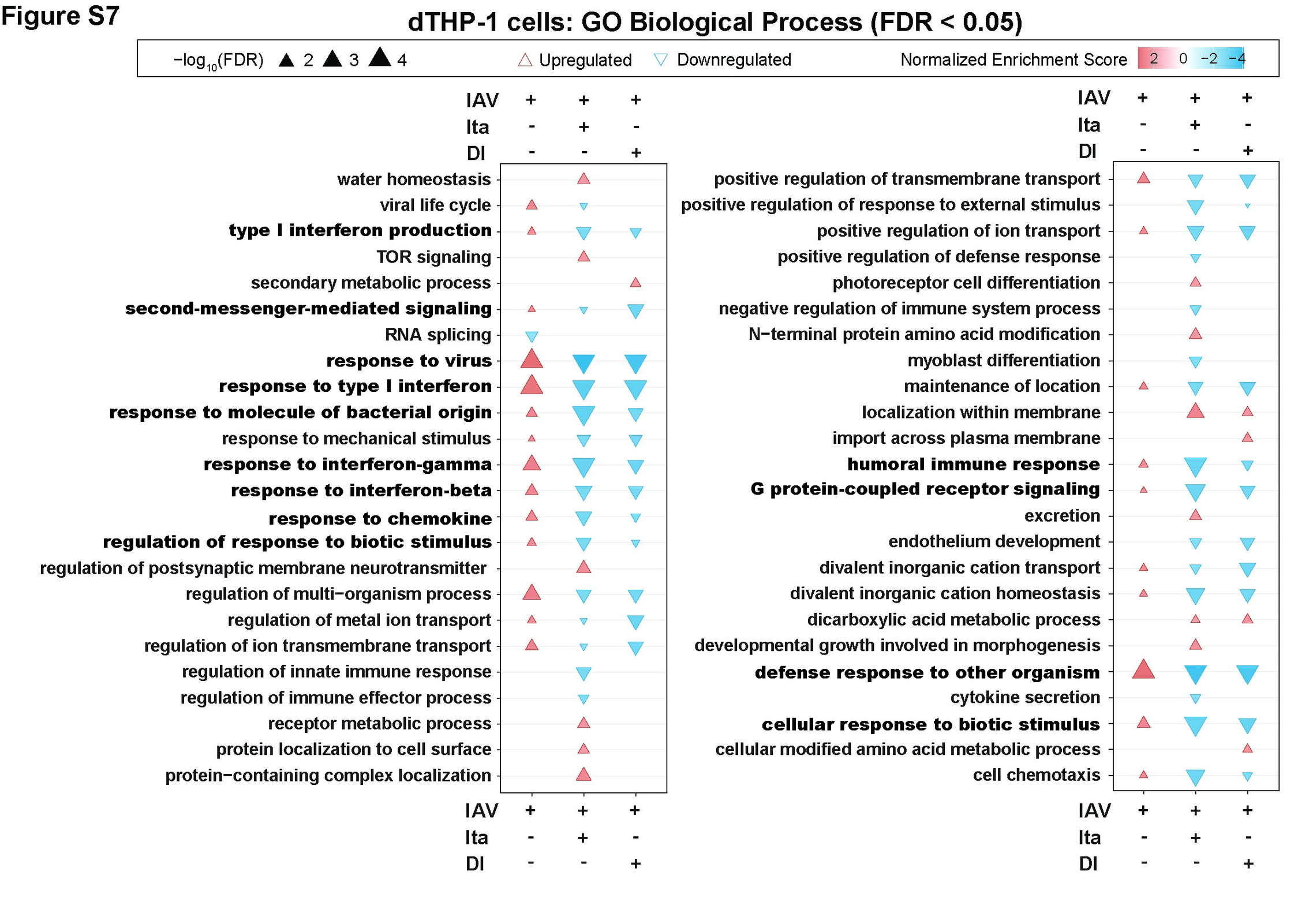

Supplement: S7 Fig — Cells were infected with IAV (PR8M, MOI = 1) and gene expression assessed by microarray analysis 12 h p.i. Enrichment analysis of gene ontology (GO Biological Process) terms based on the microarray data shown in Fig 3, using DEGs (p<0.05, FC >|1.5|) as input. GO terms are listed in reverse alphabetical order from top to bottom (page split after “protein”), with upright (red) triangles indicating enrichment (upregulation) and downward pointing (blue) triangles depletion (downregulation). Enriched GO terms (FDR ≤0.05) reveal major induction of antiviral and inflammatory responses by infection, which are decreased by both itaconate and DI treatment (most relevant inflammation and infection related GO terms are printed in bold font). (TIF) [file ppat.1010219.s007.tif]

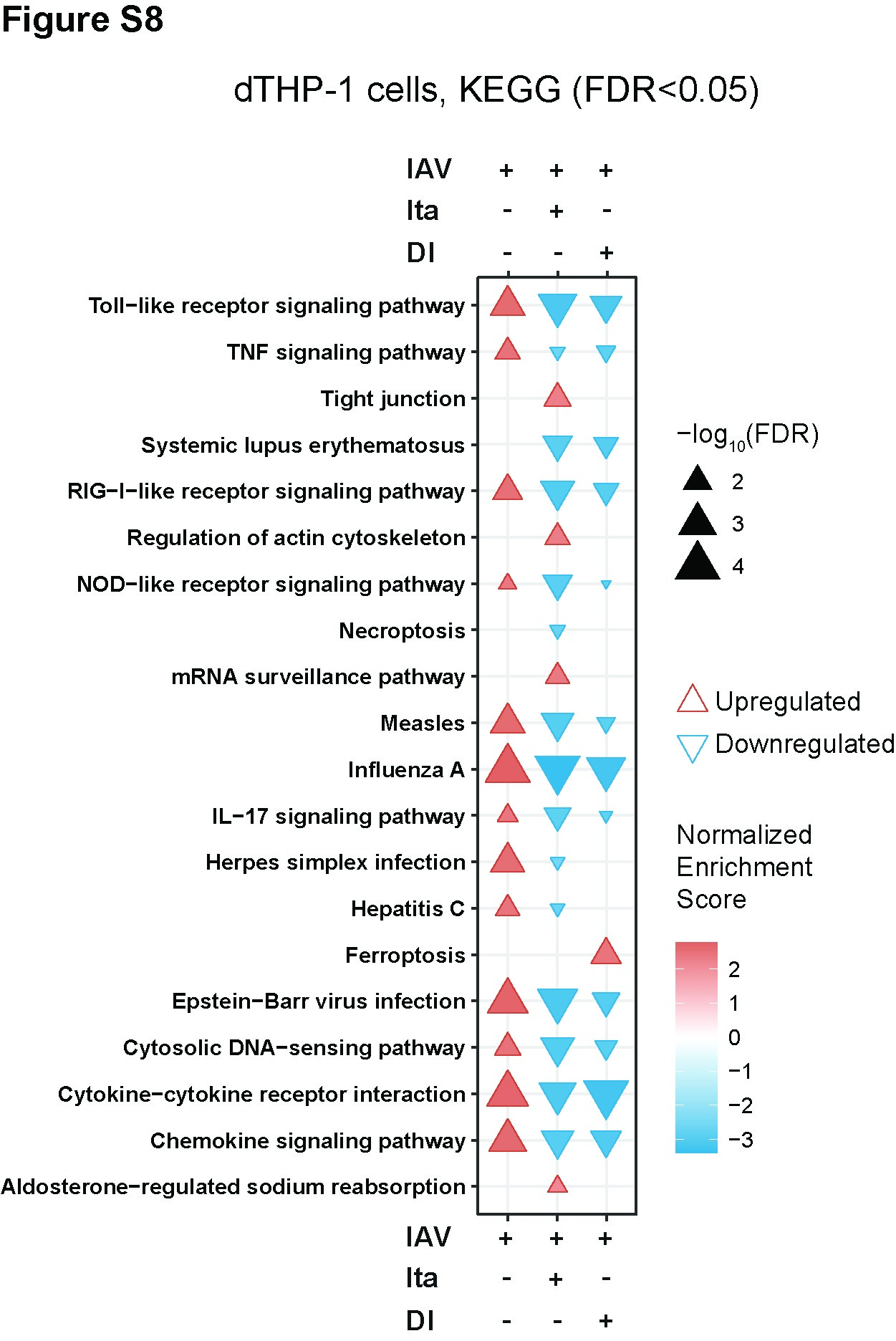

Supplement: S8 Fig — dTHP1 cells were infected with IAV (PR8M, MOI = 1) and gene expression assessed by microarray analysis 12 h p.i. Analysis based on the microarray data set used for Fig 4. A broad dampening of IFN-related and pro-inflammatory pathways by both compounds is evident. (TIF) [file ppat.1010219.s008.tif]

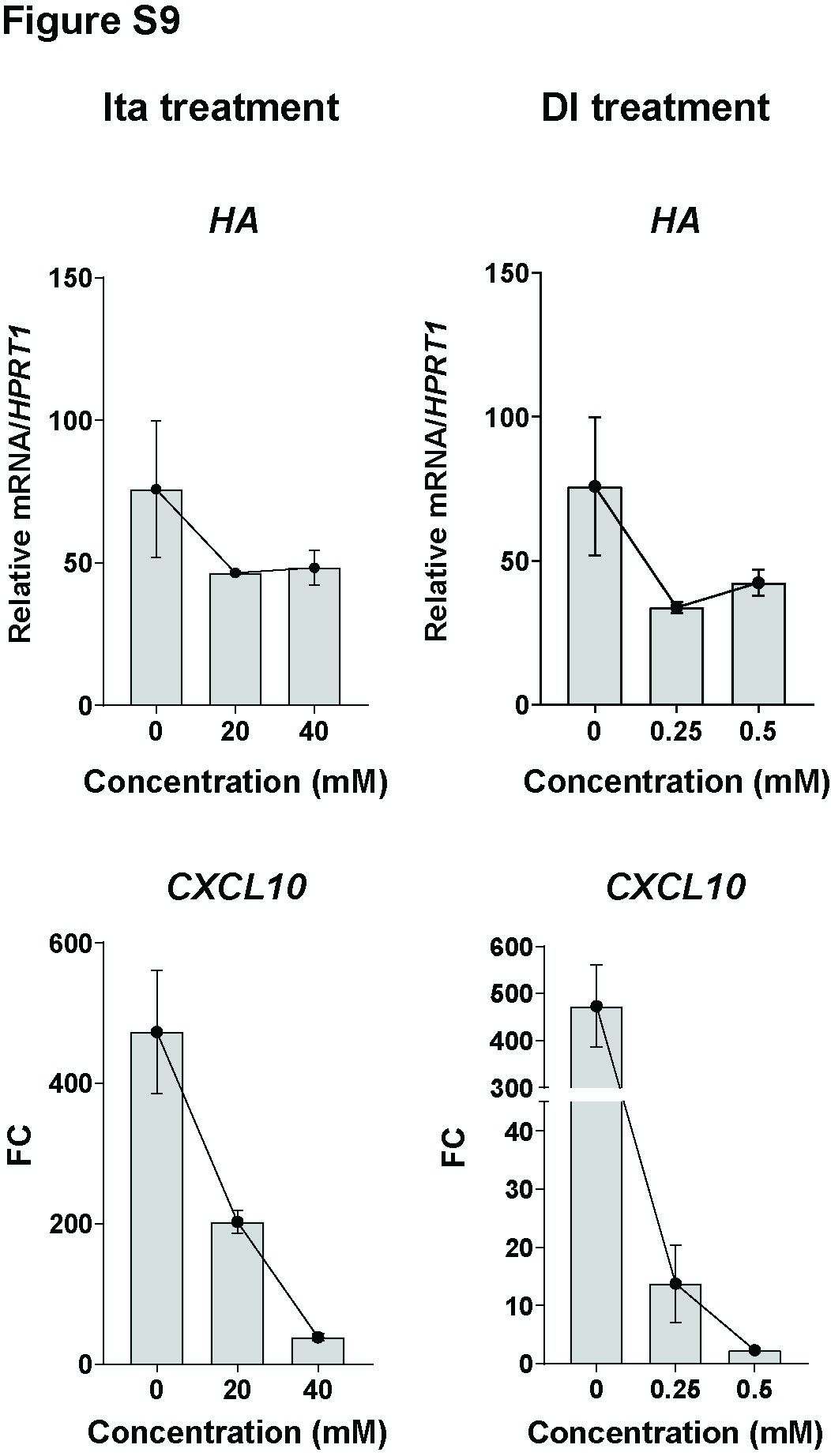

Supplement: S9 Fig — RT-qPCR using HPRT1 as reference. A549 cells were infected with IAV (PR8M, MOI = 1), and itaconate and DI treatments were given at the indicated concentrations. Expression of HA and CXCL10 mRNA was measured by RT-qPCR 24 h p.i. (n = 3). Reference for fold change = uninfected cells 24 h. DI concentrations ≥ 1 mM could not be evaluated due to widespread cytotoxicity seen by light microscopy. Toxicity data of the compounds on A549 cells are shown in S2 Fig. (TIF) [file ppat.1010219.s009.tif]

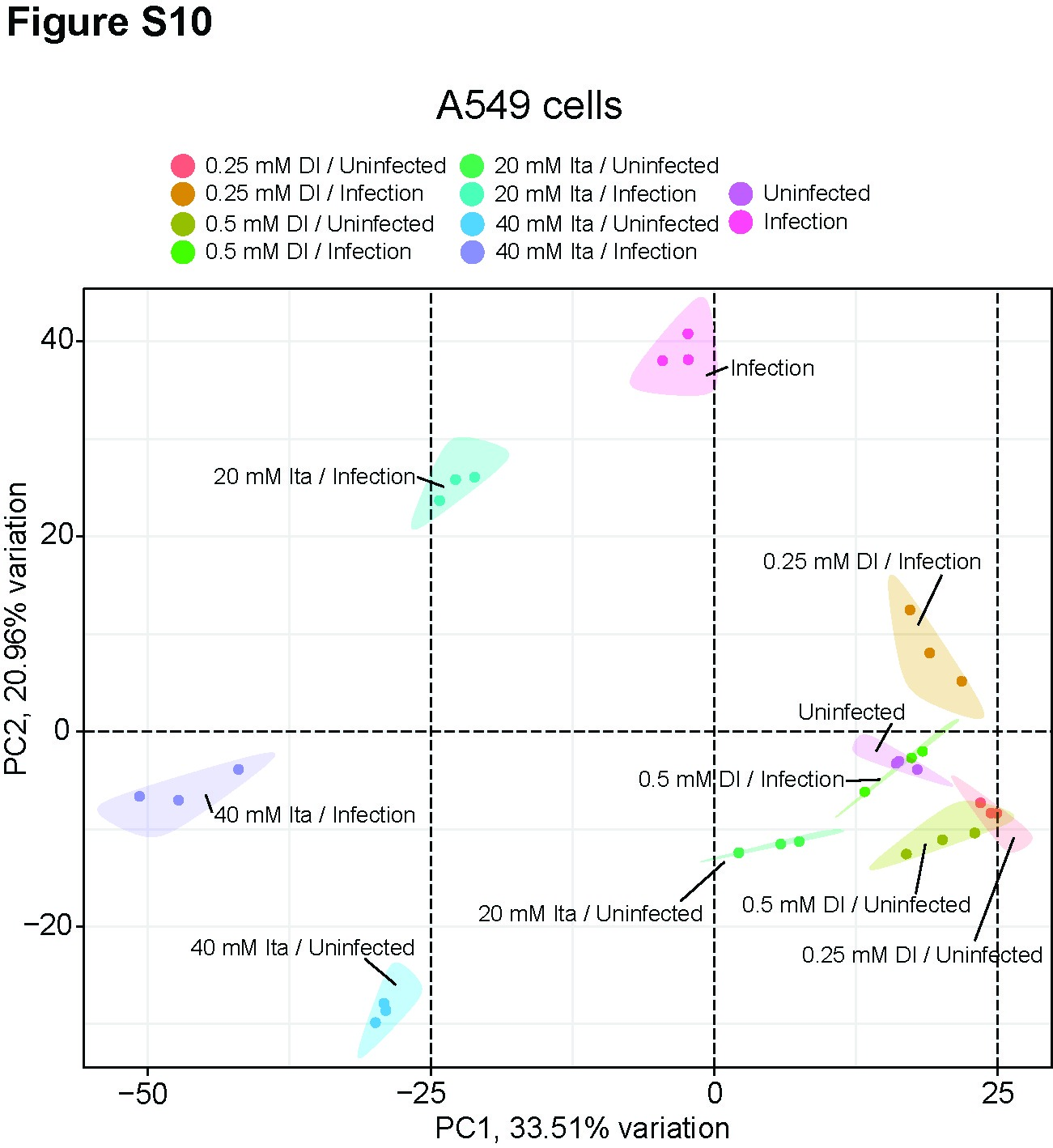

Supplement: S10 Fig — A549 cells were infected with IAV (PR8M, MOI = 1) and gene expression was assessed by microarray analysis 24 h p.i. PCA based on the microarray analysis used for S11–S13 Figs, but additionally including treatment with 0.25 mM DI. The PCA shows normalization of IAV-driven reprogramming of gene expression, but increasing impact of itaconate on cellular responses with increasing doses. Toxicity data and dose-response curves of the compounds for HA mRNA and CXCL10 mRNA expression are shown in S2 and S9 Figs, respectively. (TIF) [file ppat.1010219.s010.tif]

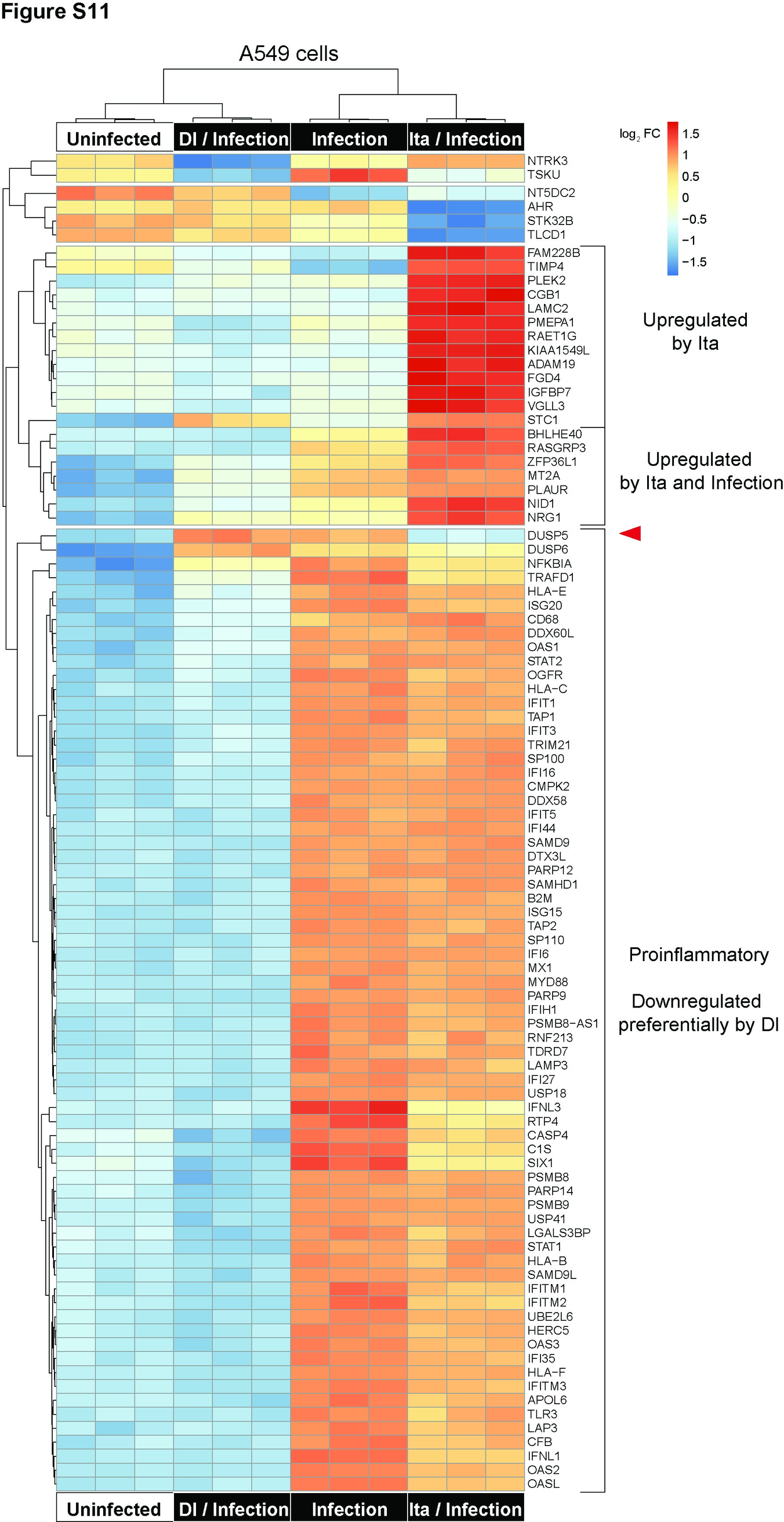

Supplement: S11 Fig — A549 cells were infected with IAV (PR8M, MOI = 1) in the presence or absence of itaconate or DI treatment, and gene expression was assessed by microarray analysis 24 h p.i. Analysis based on the microarray data used for the GO enrichment analysis in S13 Fig. Unsupervised hierarchical clustering analysis of the 100 most significant DEGs (FDR F-test <1.46E-10). DI-treated infected and uninfected cells cluster in one clade, and infected and itaconate-treated infected cells in the other. There is a large clade mostly containing pro-inflammatory genes, which are downregulated by DI, whereas effects of itaconate are much weaker. DUSP95 is exclusively downregulated by itaconate (arrowhead). There are two smaller clades of genes that form an “itaconate signature”, comprising genes that are, albeit to a lesser extent, also upregulated by IAV infection, or uniquely by itaconate. (TIF) [file ppat.1010219.s011.tif]

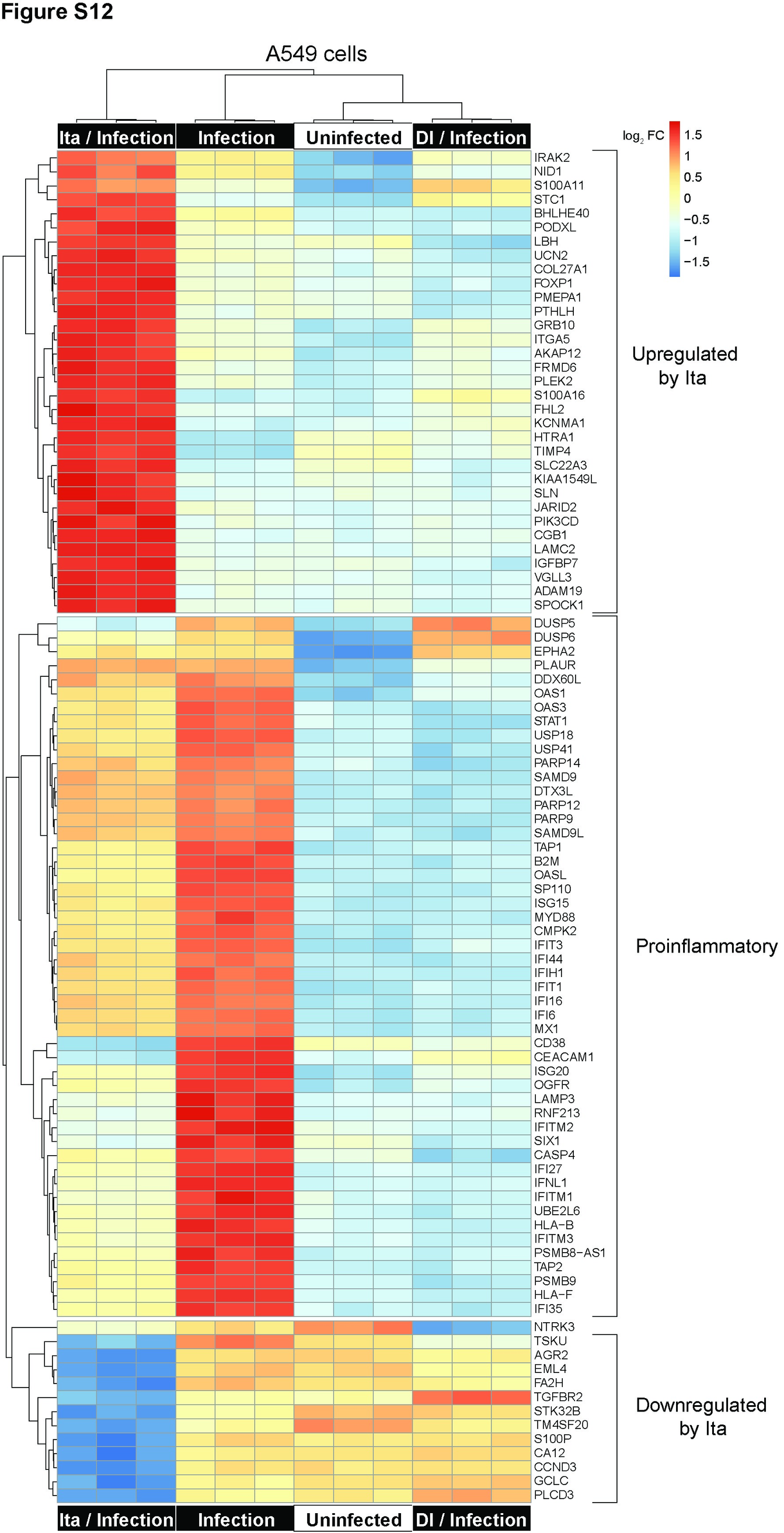

Supplement: S12 Fig — A549 cells were infected with IAV (PR8M, MOI = 1) in the presence or absence of itaconate or DI treatment, and gene expression was assessed by microarray analysis 24 h p.i. Hierarchical clustering analysis, using a subset of the microarray analysis used for the PCA in S10 Fig. Compared to the 20 mM itaconate concentration (S11 Fig), itaconate-treated IAV infection is now in a separate clade, indicating that the impact of itaconate on the cells dominates that of the infection. Downregulation of the inflammation-driven clade is now more pronounced, the itaconate-unique signature is stronger, and there also is a clade comprised of genes downregulated by IAV. (TIF) [file ppat.1010219.s012.tif]

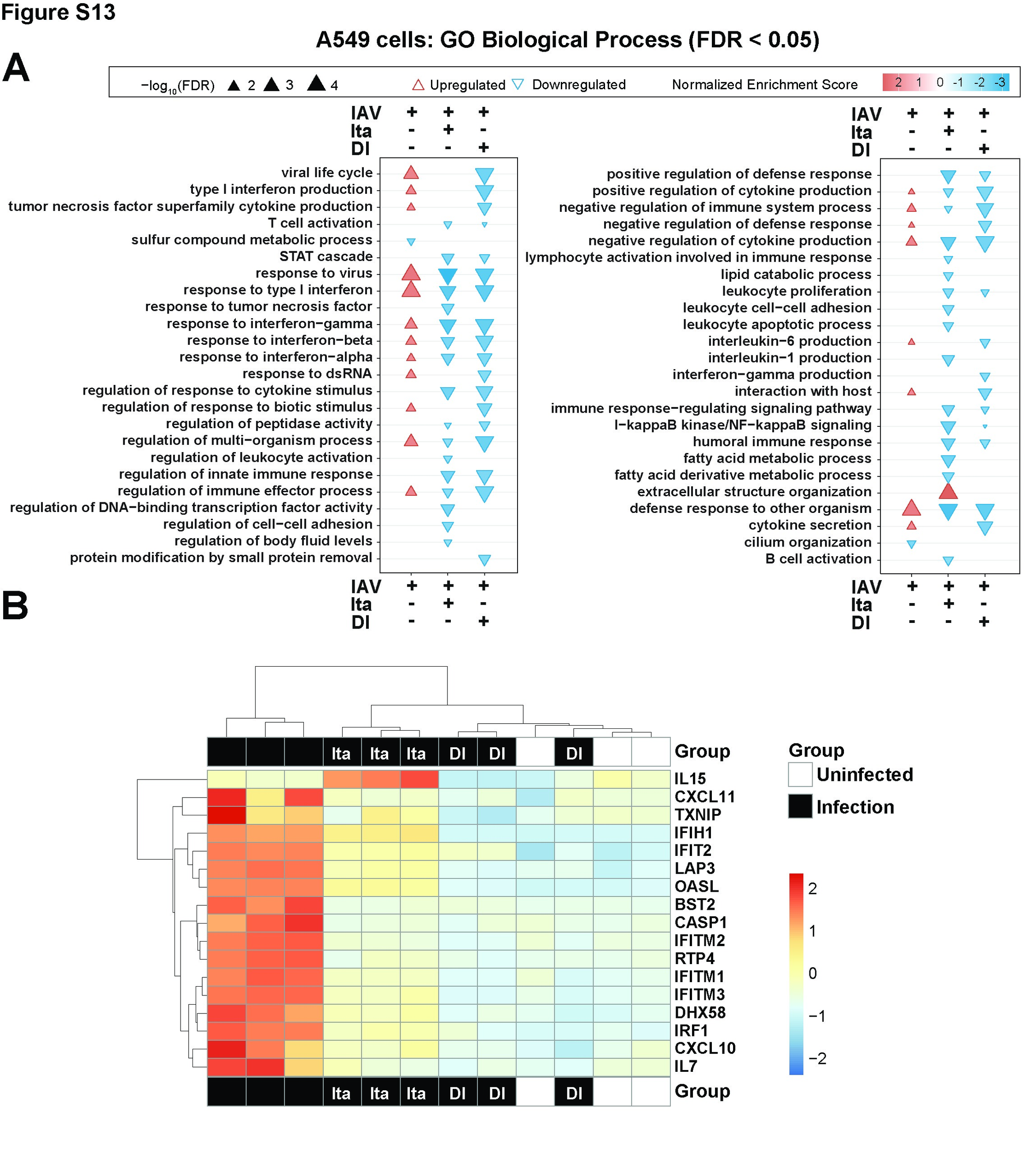

Supplement: S13 Fig — Analysis performed based on the microarray data also used for the hierarchical clustering analysis in S11 Fig. Cells were, in the case of treatment, incubated overnight with DI (0.5 mM) or itaconate (20 mM), and then infected with IAV (PR8M, MOI = 1). Gene expression was determined by microarray analysis 24 h p.i. (n = 4). A. GO enrichment (FDR <0.05) analysis using DEGs (p<0.05, FC >|1.5|) as input. GO terms are listed in reverse alphabetical order from top to bottom, with upright (red) triangles indicating enrichment (up-regulation) and downward pointing (blue) triangles depletion (down-regulation). B. Hierarchical cluster analysis illustrating downregulation of a subset of genes involved in IFNA/B signaling. (TIF) [file ppat.1010219.s013.tif]

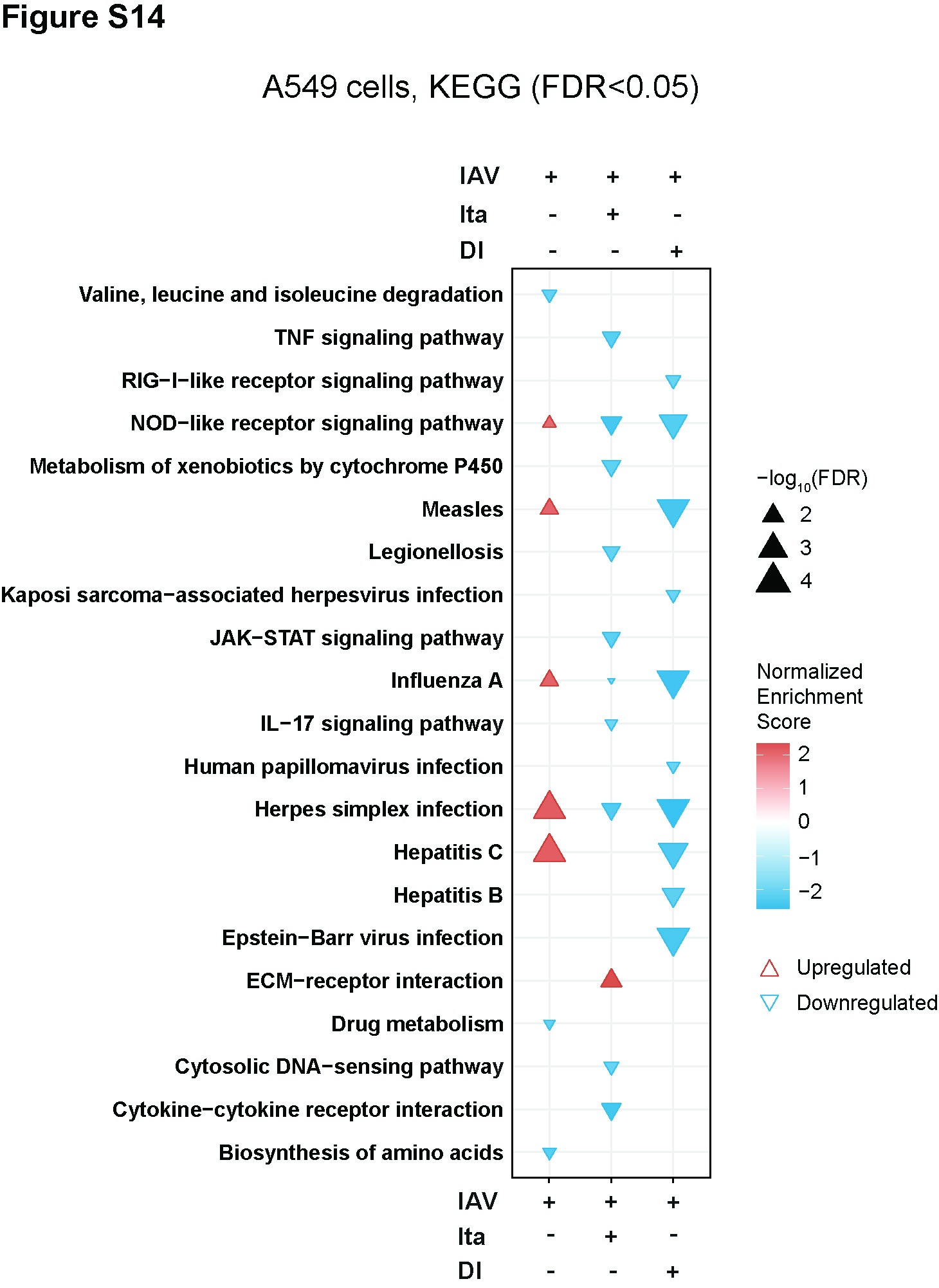

Supplement: S14 Fig — Analysis performed based on the microarray data also used for the hierarchical clustering analysis in S11 Fig. A549 cells were infected with IAV (PR8M, MOI = 1) in the presence or absence of itaconate or DI treatment, and gene expression was assessed by microarray analysis 24 h p.i. (TIF) [file ppat.1010219.s014.tif]

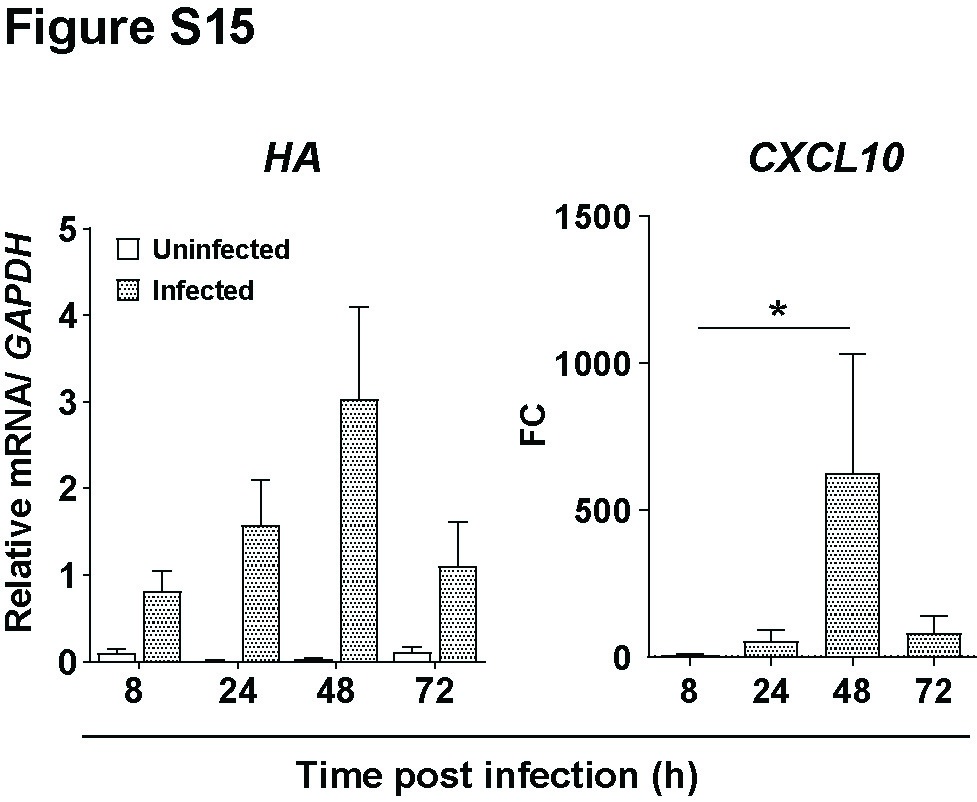

Supplement: S15 Fig — Human primary lung tissue from patients with emphysema or pulmonary arterial hypertension (n = 7 donors, 3 tissue pieces per donor per treatment) was infected with IAV (PR8M, MOI = 1) for 72 h and HA and CXCL10 mRNA expression measured by RT-qPCR, using HPRT1 as internal reference. Reference for fold change = uninfected tissue at the same time point. *p<0.05; **p<0.01; ***p<0.001 (Mann-Whitney U test). (TIF) [file ppat.1010219.s015.tif]

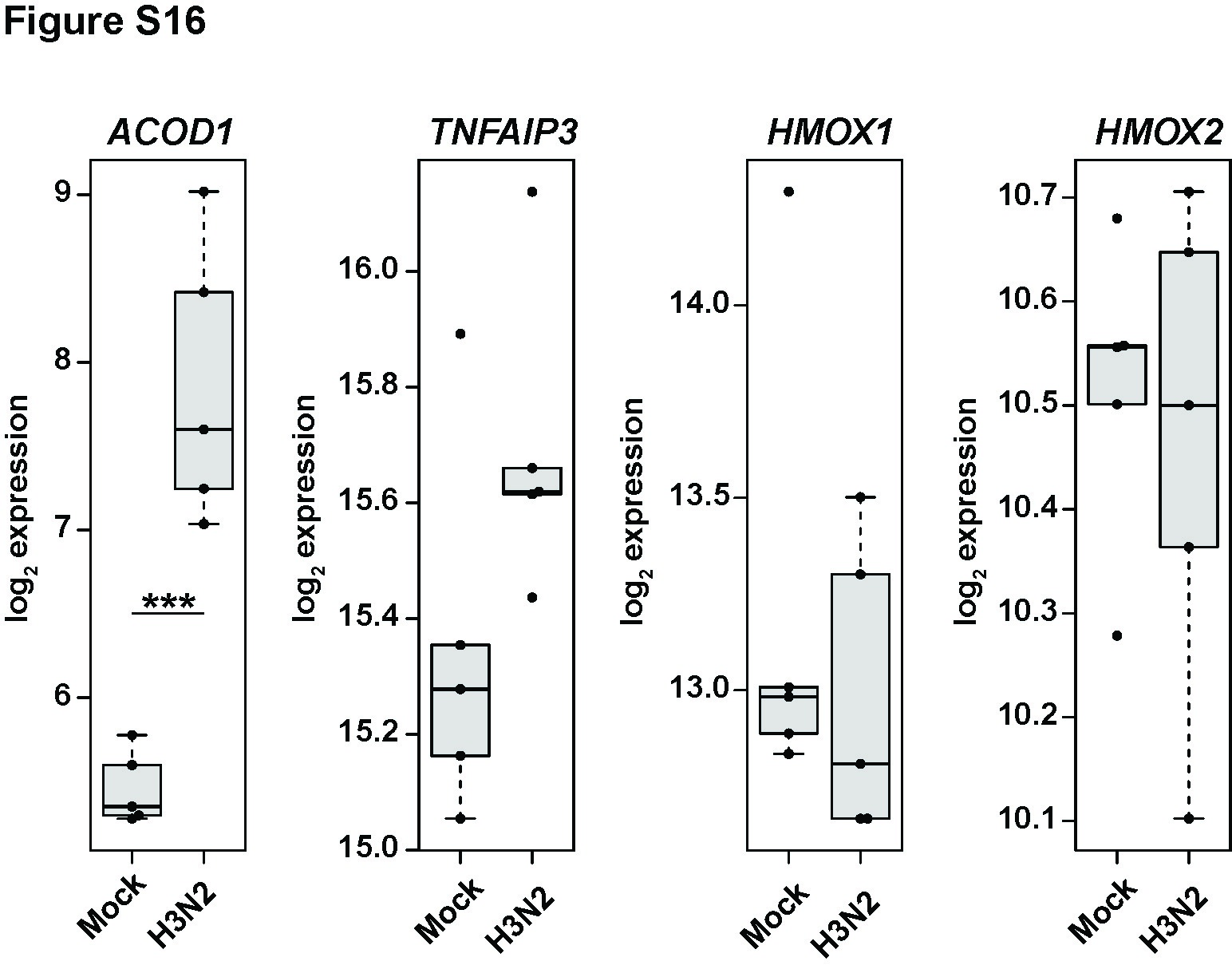

Supplement: S16 Fig — Reanalysis of a published dataset of gene expression (RNAseq) in IAV A/Panama/2007/1999 (H3N2) infection (dose not specified) of human lung tissue derived from tumor-free margins obtained during lobectomy for lung carcinoma (39). A strong induction of ACOD1 expression and a tendency towards increased TNFAIP3 expression are seen. As opposed to the strong induction of both genes in the mouse model (Fig 1A), HMOX1 and HMOX2 expression is unchanged. *p<0.05; **p<0.01; ***p<0.001 (pairwise t-tests with pooled SD). (TIF) [file ppat.1010219.s016.tif]

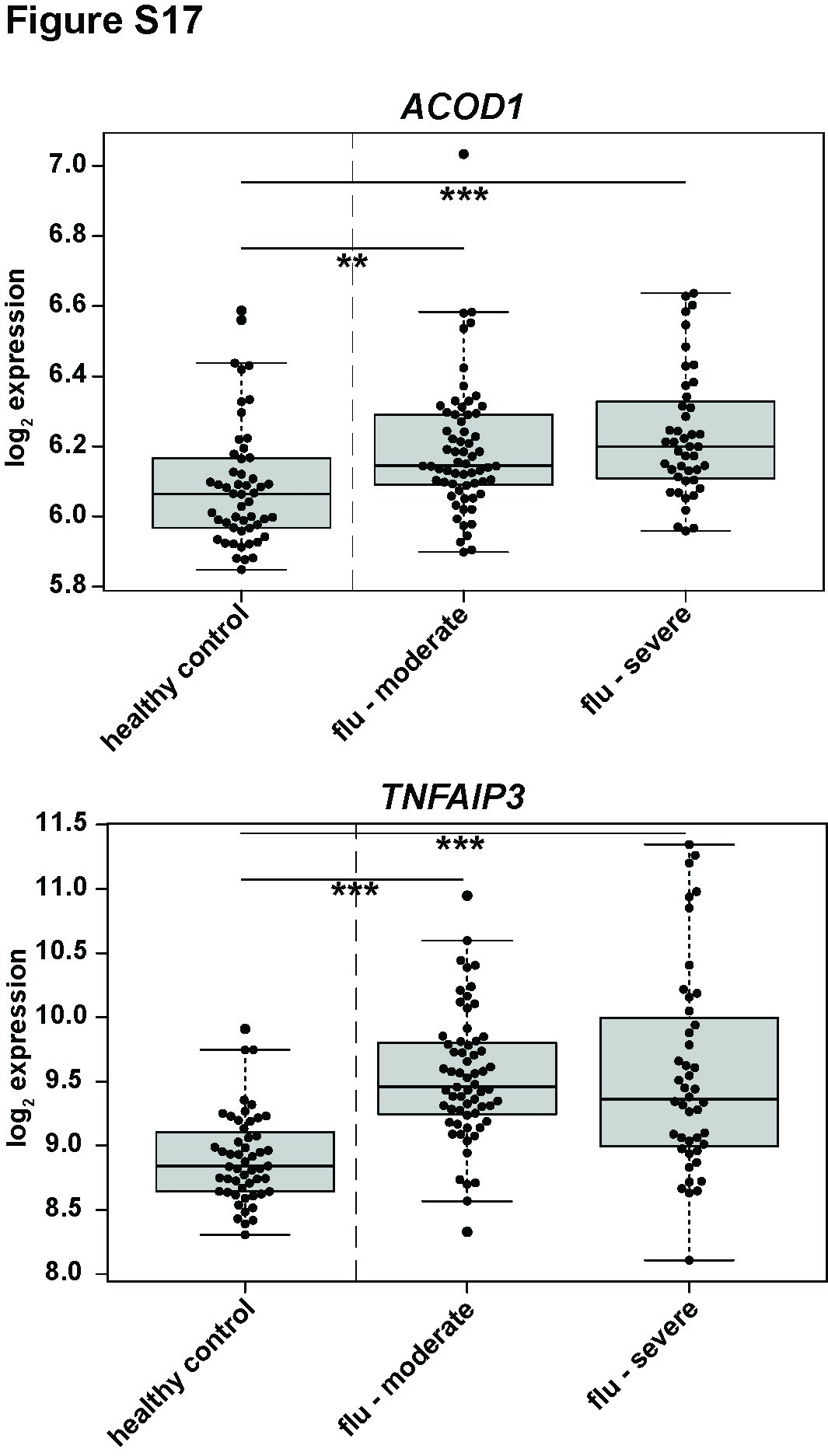

Supplement: S17 Fig — Reanalysis of a published data set of gene expression in whole blood from patients with moderate and severe influenza and healthy controls [64]. *p<0.05; **p<0.01; ***p<0.001 (pairwise t-tests with pooled SD). (TIF) [file ppat.1010219.s017.tif]

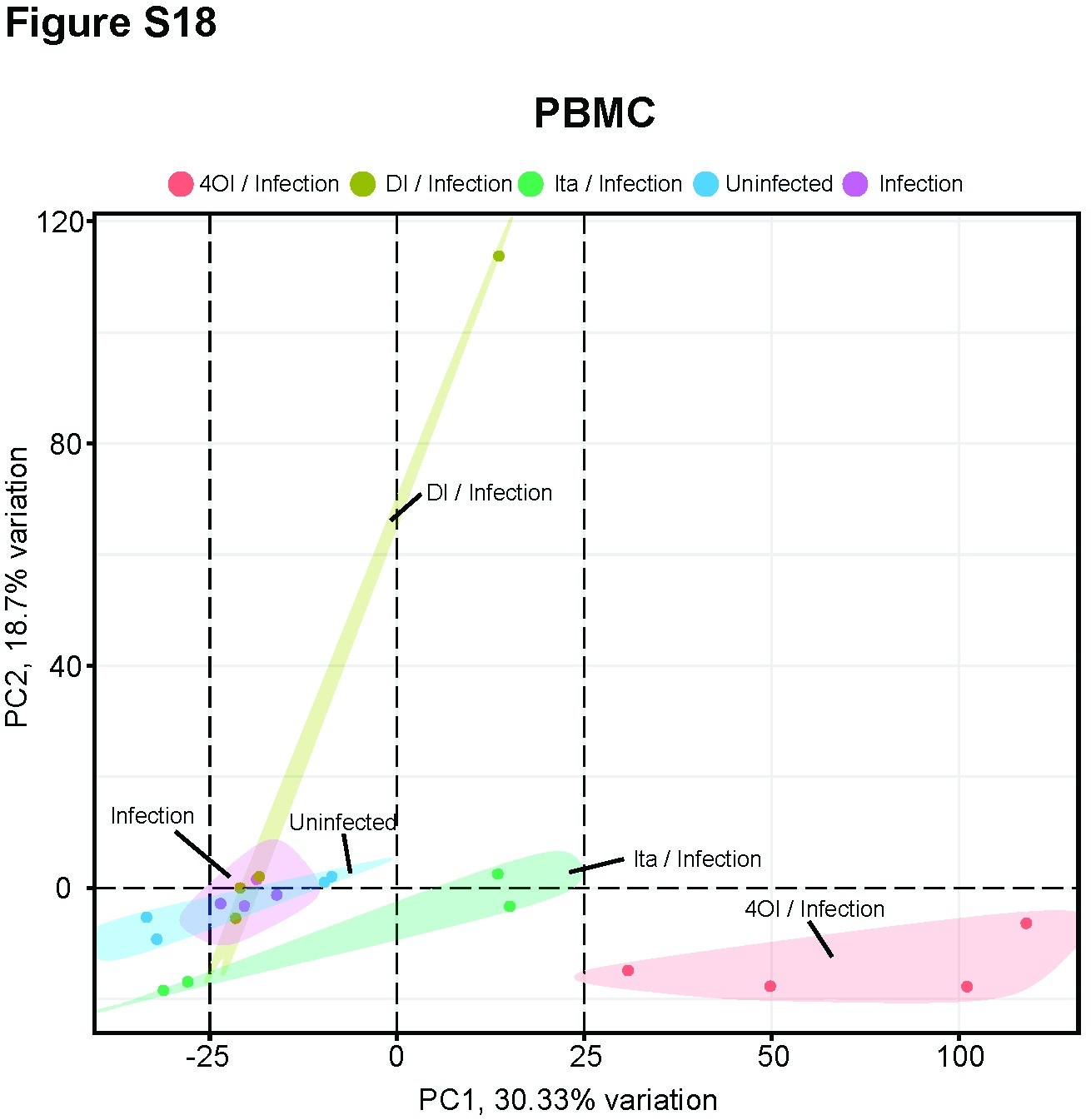

Supplement: S18 Fig — Analysis of mRNA expression in a subgroup (n = 4 donors) of the PBMC samples that were used for the targeted assays shown in Fig 8. PBMC were infected with IAV (PR8M, MOI = 1) and gene expression was analyzed by microarray analysis 12 h p.i. A PCA was performed on the same microarray data as used for the GO enrichment analysis in Fig 8C. There is a less pronounced effect of IAV infection (control and IAV infected samples are not clearly separated) than on dTHP-1 and A549 cells, but pronounced additional broad changes in gene expression due to treatment with 4OI, itaconate (note two outliers), and DI (note one outlier) are seen. (TIF) [file ppat.1010219.s018.tif]

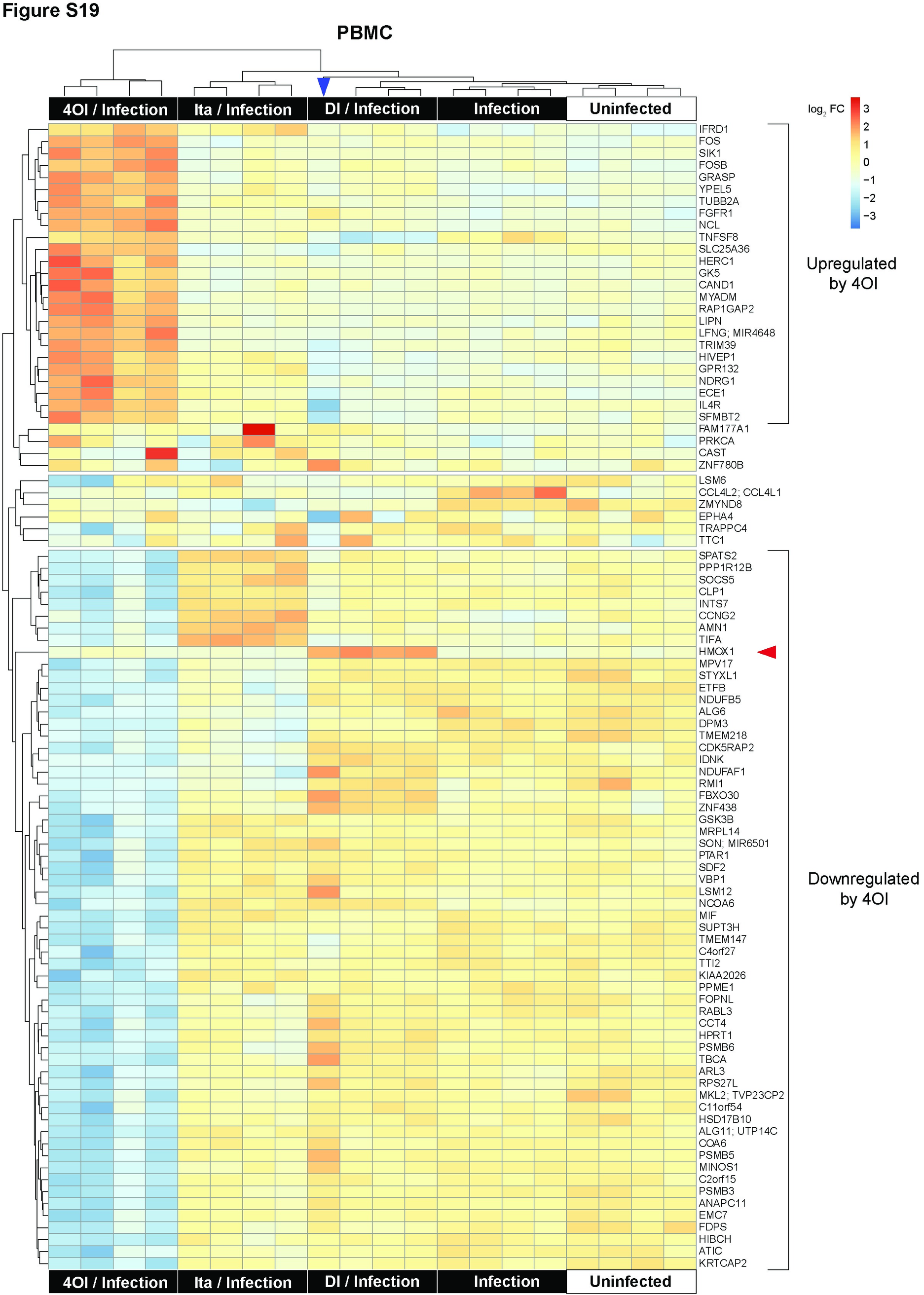

Supplement: S19 Fig — PBMC were infected with IAV (PR8M, MOI = 1) and gene expression was analyzed by microarray analysis 12 h p.i. Analysis based on the same microarray data as Figs 8C and S18. The 100 most significant DEGs were selected (FDR F-test <3.72E-05). Transcriptome changes are mostly due to marked effects of 4OI (more down- than up-regulation) on genes that are not affected by IAV infection, indicating general effects on cell homeostasis. However, induction of HMOX1 by DI in IAV infection is evident (red arrowhead). The blue arrowhead points to the extreme outlier under DI treatment seen in the PCA (S18 Fig). (TIF) [file ppat.1010219.s019.tif]

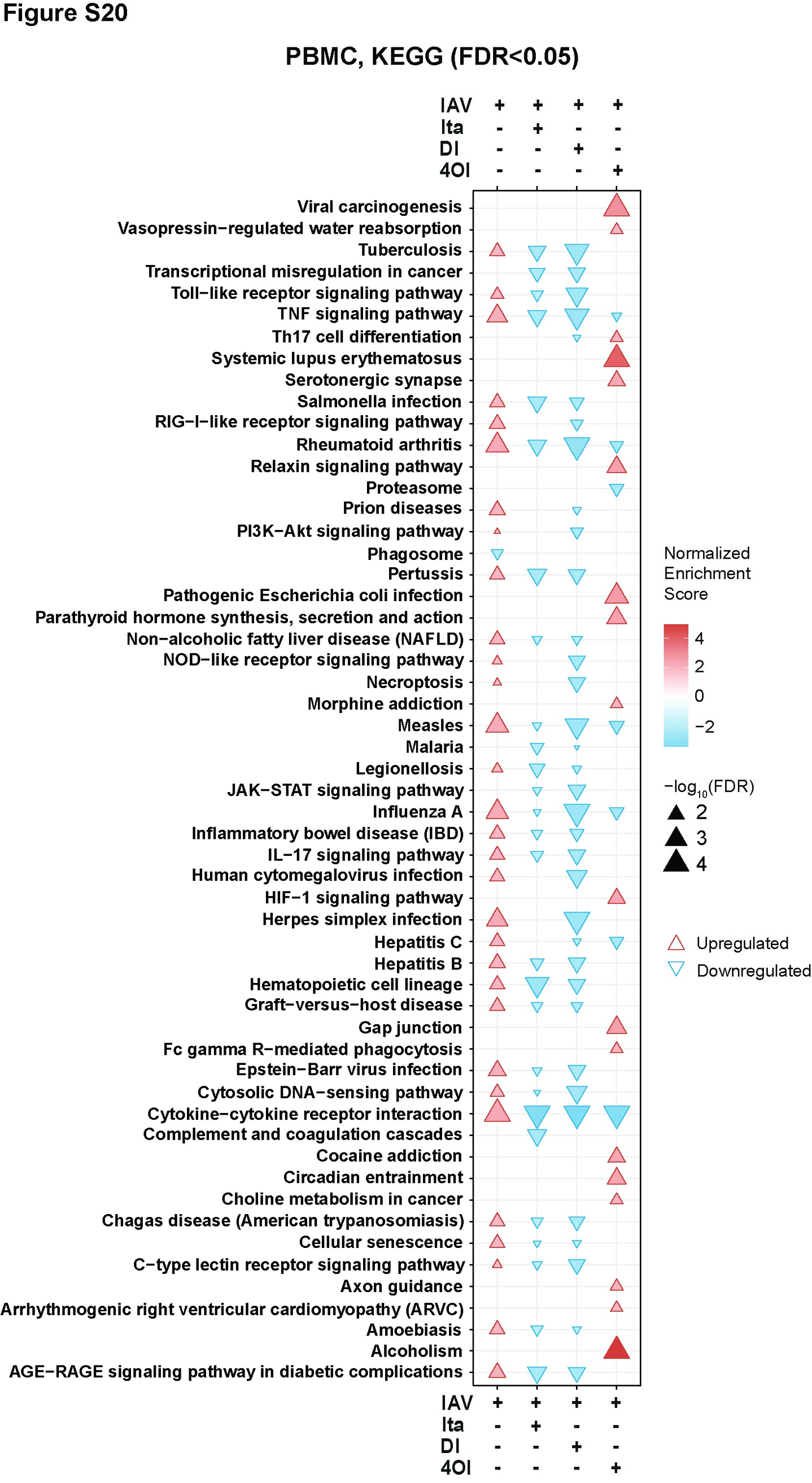

Supplement: S20 Fig — PBMCs were infected with IAV (PR8M, MOI = 1) and gene expression was analyzed by microarray analysis 12 h p.i. Analysis based on the same microarray data as S18 and S19 Figs. KEGG terms with an FDR <0.05 in at least one group were selected. The threshold for inclusion in the chart was raised to FDR <0.01 if a pathway was enriched/depleted only in a single treatment and not by IAV infection alone (e.g., Alcoholism in 4OI treatment). (TIF) [file ppat.1010219.s020.tif]

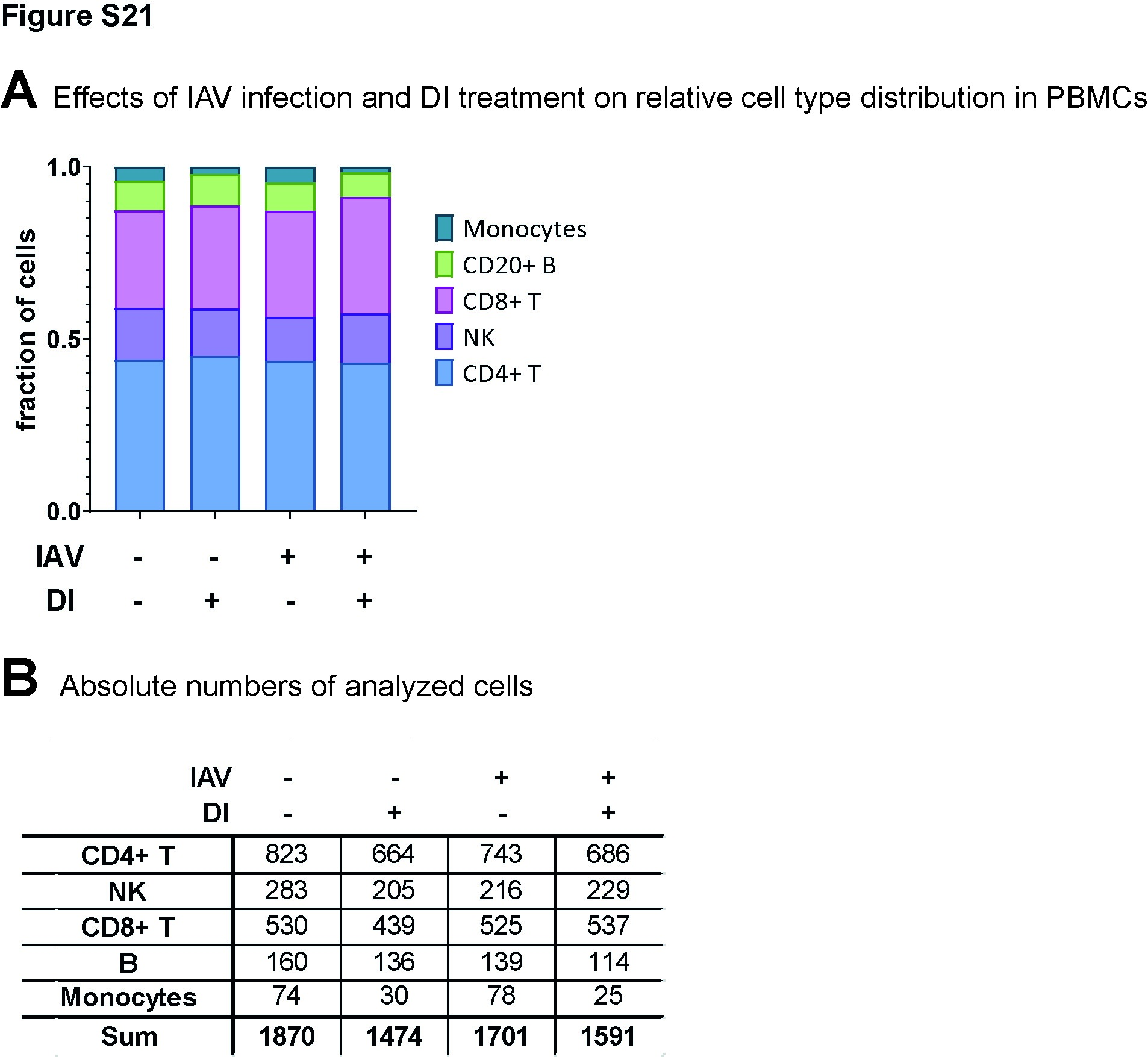

Supplement: S21 Fig — PBMCs were infected with IAV (PR8M, MOI = 1) and gene expression was assessed by scRNAseq 12 h p.i. Analysis based on the data set derived from the experiment shown in Fig 9. A reduction of CD14+ monocytes is apparent under DI treatment of both uninfected and infected PBMC. A. Percentages. B. Absolute numbers. (TIF) [file ppat.1010219.s021.tif]

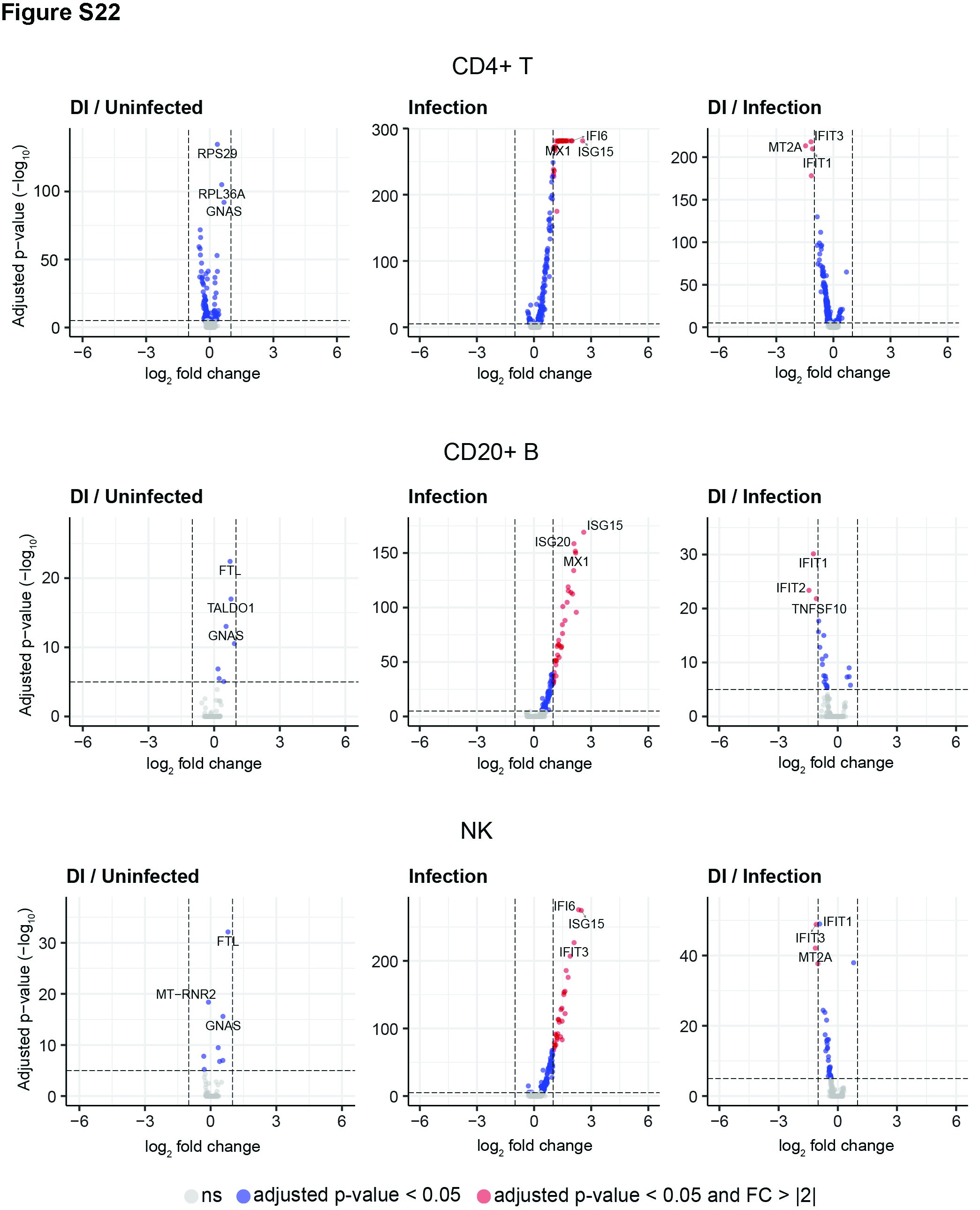

Supplement: S22 Fig — PBMCs were infected with IAV (PR8M, MOI = 1) and gene expression was assessed by scRNAseq 12 h p.i. Analysis based on the data set derived from the experiment shown in Fig 9. Volcano plots comparing effects of DI treatment, with and without IAV infection, on CD4+ cells, NK cells, and B cells. (TIF) [file ppat.1010219.s022.tif]

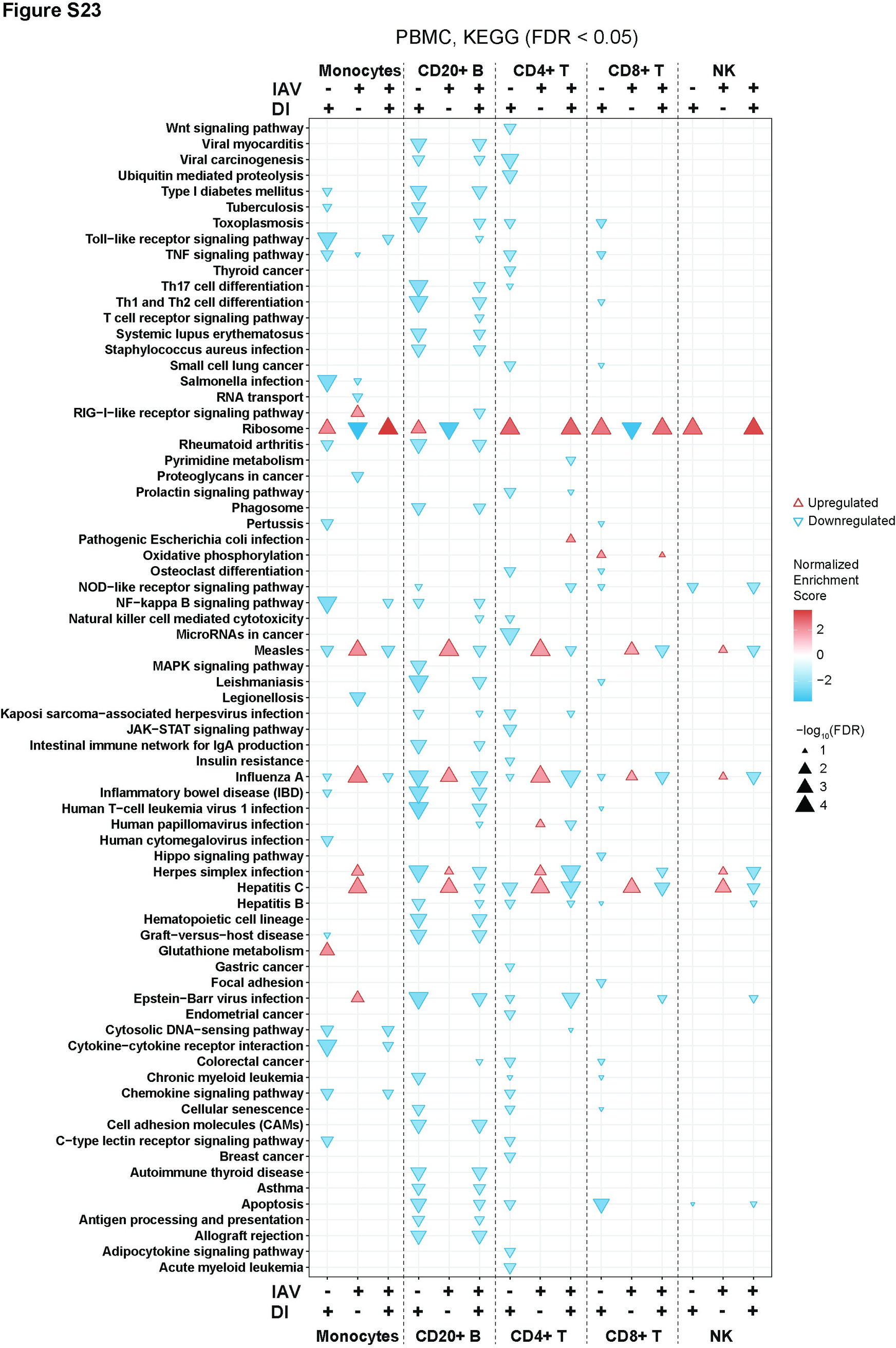

Supplement: S23 Fig — PBMCs were infected with IAV (PR8M, MOI = 1) and gene expression was assessed by scRNAseq 12 h p.i. Analyses based on the scRNAseq data used for Figs 9 and 10. Pathways that are enriched (FDR <0.05) in at least one cell type are shown. (TIF) [file ppat.1010219.s023.tif]

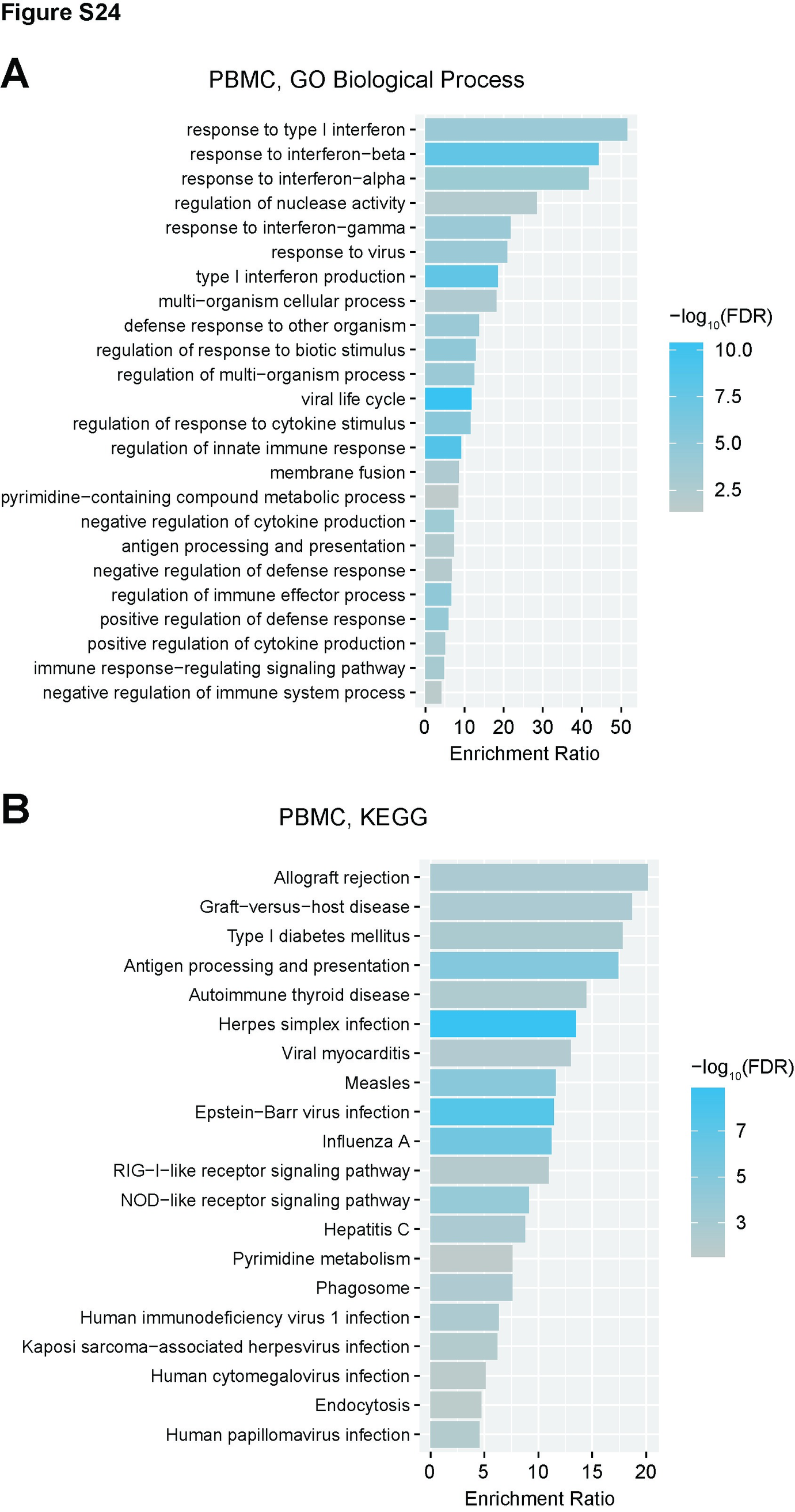

Supplement: S24 Fig — PBMCs were infected with IAV (PR8M, MOI = 1) and gene expression was assessed by scRNAseq 12 h p.i. GO Biological Process (A) and KEGG (B) functional enrichment analyses of all genes commonly differentially expressed in monocytes, CD4 and CD8 T cells, B cells, and NK cells in response to IAV infection and DI treatment (i.e. the central intersect of the Venn diagram in Fig 9G). (TIF) [file ppat.1010219.s024.tif]

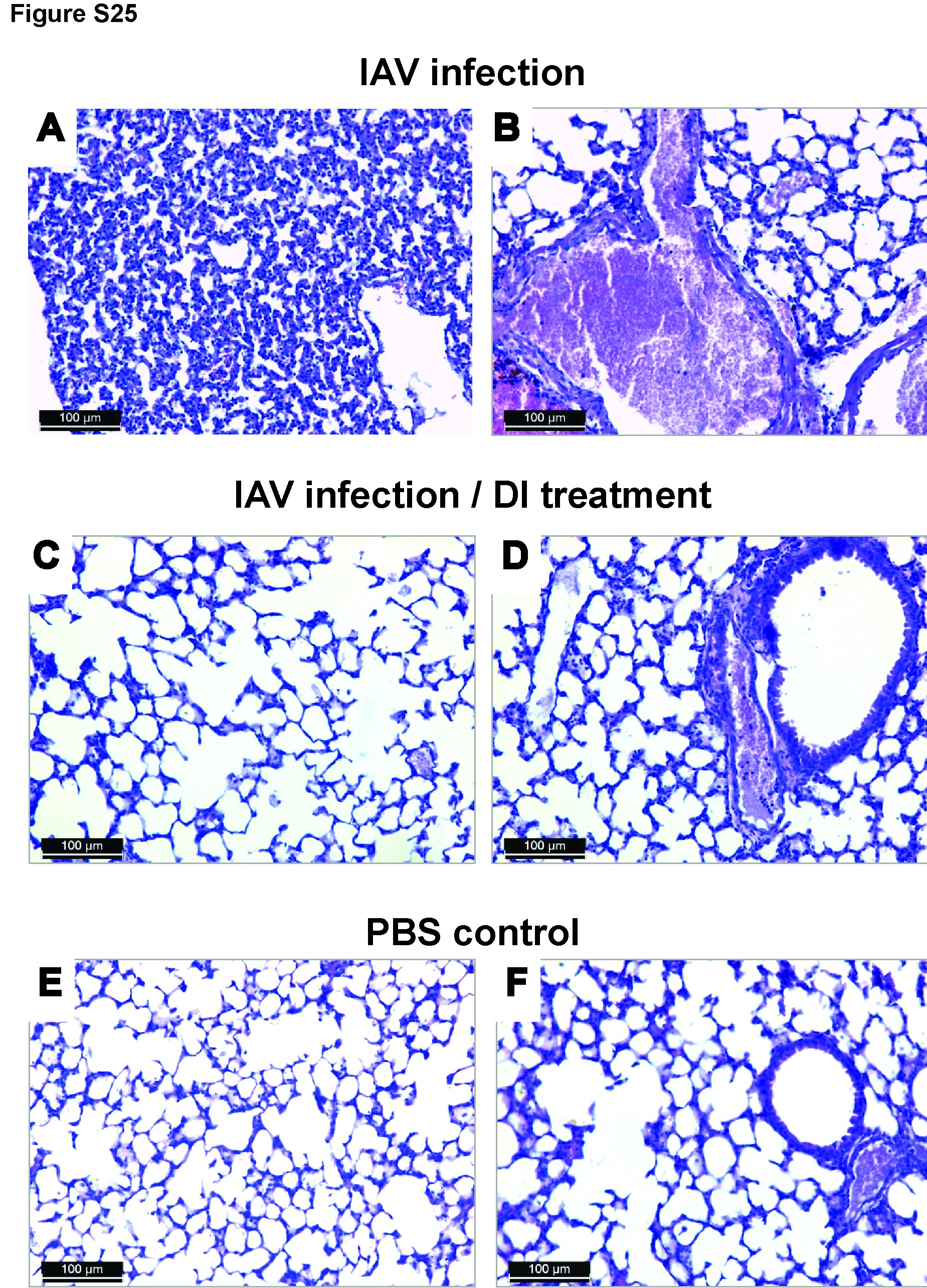

Supplement: S25 Fig — Representative histological findings in lungs of IAV infected mice with or with DI treatment (50 mg/Kg once daily, IP) compared with mock-infected (PBS control) mice. Representative photomicrographs (H&E stains) of sections used for the inflammation score analysis shown in Fig 11F. A,B. IAV infection without treatment. A. Massive inflammatory infiltration with neutrophil predominance, which fills the alveolar air spaces. B. Necrotizing bronchiolitis. There is necrosis of the bronchiolar wall, with submucosal edema and vascular congestion. The epithelial layer is desquamating, and necrotic epithelial cells are present in the lumen. Interstitial fibrosis with thickening of the muscular artery wall is present throughout. C,D. IAV infection with DI treatment. C. Normal appearing alveoli with preserved air spaces and no inflammatory infiltrates. D. Bronchiole without inflammation, but thickening of the muscular artery wall. E,F. Mock infection (PBS control). E. Normal appearing alveoli with preserved air spaces and no inflammatory infiltrates. F. Normal appearing bronchiole without signs of inflammation or wall thickening. (TIF) [file ppat.1010219.s025.tif]
