# Supplementary material for: Itaconate and derivatives reduce interferon responses and inflammation in influenza A virus infection
Source: PLoS Pathog. 2022 Jan 13;18(1):e1010219. doi: 10.1371/journal.ppat.1010219 (PMC8846506; doi:10.1371/journal.ppat.1010219)
Supplement: Attachment — Submitted filename: Pessler_influenza.pdf [file ppat.1010219.s026.pdf]

The manuscript by Sohail and colleagues investigated the inflammatory response of itaconate and itaconate derivatives upon infection of influenza A virus. The authors treated itaconate and derivatives to cells infected with the virus and observed that interferon response was repressed without affecting viral replication. Further, they compared gene expression in treated cells and identified gene signatures induced by each compound (i.e. itaconate, DMI, and 4OI). The findings may provide useful information on the complex inflammatory response upon virus infection and how itaconate and its derivative influence inflammation linking itaconate molecules to inflammatory response and disease outcome.

The impact of itaconate and chemical derivatives on the immune response is quite interesting. However, the specific mechanism at play and the distinct effect of itaconate and chemical derivatives are not clear. Recent studies have demonstrated that itaconate and itaconate derivatives have a distinct impact on metabolism, signaling (i.e. Nrf2), and immune response, specifically recent work by the Artyomov lab (PMID: 32694786). As such, itaconate and itaconate derivatives should not be used interchangeably and might be better discussed as different compounds. Some additional studies would help to clarify these issues, as the overall impact on inflammation is strong.

1. The authors stated that "*chemical variants of itaconate, rather than its native form, will take the lead in further translational development to the bedside*" (line 428). The "cytoprotective properties" and the mechanism of action of itaconate and chemical derivatives are not well understood. It became clear that itaconate and itaconate derivatives have very different impacts on metabolism (specifically SDH inhibition, see Artyomov lab PMID: 32694786). As such, the primary mechanism of action is not well understood. At times, the authors may reframe their statements; for instance, "both compounds likely exert cytoprotective effects by reducing ROS generation via inhibition of SDH." (line 102).

2. Interestingly, D2 mice models have decreased ACOD1 expression compared to B6 mice. Does reduced ACOD1 expression led to reduced itaconate levels in the D2 animal model? Expression level does not reflect metabolite level, and itaconate concentrations might be similar in both animal models. If itaconate levels are identical, ACOD1/itaconate might not be the primary driver for the different disease severity described in the two animal models. Further, some quantification of intracellular itaconate levels would be beneficial to better understand the treatment doses chosen for this study as 25 mM or 40 mM extracellular itaconate seems to be very high. Are those concentrations physiological relevant? Specifically, which itaconate concentrations are achieved in macrophages and PBMCs upon infection (Fig. 2)?

3. Itaconate derivatives seem to decrease ACOD1 expression levels (Fig 8). Some effects observed by treatments with itaconate derivatives might be due to decreased endogenous itaconate synthesis. On the other hand, 4OI might be converted into itaconate (Hooftman et al. 2020, study with 13C 4OI). How do itaconate levels change in response to DMI and 4OI treatments? Further, the authors may want to repeat some key experiments in IRG1KO cells treated with DMI or 4OI to decipher the impact of itaconate derivatives compared to endogenous itaconate levels.

4. The authors pretreated cells for 24h before infection (Fig 3) for most of their studies. Are similar results achieved with acute treatments (as opposed to pretreatments)? Pretreatments might be clinically challenging.

   Minor:
   The authors may cite and discuss some key studies on itaconate derivatives, specifically work by Artyomov Lab (PMID: 32694786, impact on inflammation), Hooftman et a (PMID: 32791101, Cell Metab, inhibition of NLRP2 inflammasome activation by 4OI), and Sethy et al. (2019, J.Med.Chem, identification of itaconate derivates as potential anti-influenza agents). For instance, the current manuscript demonstrated that CXCL10 expression was decreased upon itaconate/derivative treatment, while work by Artyomov lab reported increased levels upon itaconate treatment in IRG1KO conditions.
