# Supplementary material for: Itaconate and derivatives reduce interferon responses and inflammation in influenza A virus infection
Source: PLoS Pathog. 2022 Jan 13;18(1):e1010219. doi: 10.1371/journal.ppat.1010219 (PMC8846506; doi:10.1371/journal.ppat.1010219)
Supplement: Attachment — Submitted filename: Response file Sohail et al.docx [file ppat.1010219.s027.docx]

| **Reviewer** | **Response** |
| --- | --- |
| **Part I Summary** | |
| **Reviewer 1** | **Response/action taken** |
| Recent studies have demonstrated that itaconate and itaconate derivatives have a distinct impact on metabolism, signaling (i.e. Nrf2), and immune response, specifically recent work by the Artyomov lab (PMID: 32694786). As such, itaconate and itaconate derivatives should not be used interchangeably and might be better discussed as different compounds. Some additional studies would help to clarify these issues, as the overall impact on inflammation is strong. | We have tried to clarify this concept in Introduction (lines 104-111 ff, specifically considering (Swain, Bambouskova et al. 2020) and (Bambouskova, Potuckova et al. 2021) in Introduction and Discussion.  E.g., lines 105-114, 469-471  We also performed several new experiments which all support the notion that the compounds have both common and distinct features:  P-AKT inhibition (DI and 4OI, but not Ita; Fig. 6), P-STAT1 inhibition (OI>DI/Ita, Fig. 6)  Cytokine release from DI vs OI treated dTHP1 cells (Fig. S5),  effects of preincubation with Ita vs. OI (Fig. 4), and inhibition of viral release from A549 cells (4OI and IA, but practically not DI; Fig. 11).  A comparison of major features of the compounds is also summarized in a new table.  Table 1. |
| **Reviewer 2** |  |
| (no concerns) |  |
| **Reviewer 3** |  |
| The study as presented is overly descriptive and utilizes several different cell lines in vitro to perform transcriptomic analysis on each cell line following treatment with Ita and DI. There is no attempt made to understand even at a very superficial level as to what the physiological consequences are of treatment with Ita or DI. CXL10 and CCL2 are potent chemoattractants of monocytes and other myeloid as well as T cells. The authors should attempt to at the very least perform these studies in vivo in the mouse model and use flow cytometry to determine how the inflammatory response is affected by these treatments and if downregulation of CXCL10 and CCL2 transcripts matches actual reduction in protein production and a concomitant decrease in myeloid cell (monocytes, neutrophils etc) recruitment. | We appreciated the reviewer’s very constructive recommendation to study DI efficacy in an in vivo model. We were able to perform two DI treatment experiments of IAV-infected WT mice, because the coauthors in Cairo (M.T., AM) had an active animal research protocol for DI treatment. These experiments revealed a global reduction of inflammation in the lungs of treated mice. The reduction was so strong at the histological level that, in our opinion, an in-depth analysis by flow cytometry would not tell us much more than what is seen histologically, i.e. that DI exerts a remarkable, broad anti-inflammatory effect. Considering this, it might also be difficult to obtain permission from the regulatory authorities to use animals for such additional experiments.  Another important finding of these in vivo experiments is that treatment greatly reduced lung inflammation but did not affect viral titers, whereas survival was significantly greater in the treated mice (Fig. 11). Surprisingly, there was no effect on weight loss.  Considering the remarkable reduction of viral titers by 4OI in A549 cells (Fig. 11 ), greater anti-inflammatory effects than DI on dTHP1 cells (Fig. S5), and lack of effect of DI on weight loss of infected mice, we have decided to deprioritize further work on DI and to shift the focus of further in vivo (as well as in vitro) to 4OI and related molecules. However, that work required submission of a new animal research application to the regulatory authorities. Resources for this work are available only at the partner site in Braunschweig, Germany. Since it usually takes several months to obtain approval in the German regulatory environment, it is not feasible to include in vivo results of 4OI treatment in the current paper.  Reduction of CCL2 expression and macrophage recruitment by DI in vivo have already been described (Wang, Li et al. 2020). |
| In addition, macrophages are important players in regulating and mediating the inflammatory process after influenza infection in the lungs. As demonstrated by the investigators Acod1 gene expression is particularly high in M2 macrophages. There are many subsets of macrophages in the lungs that mediate distinct functions, the investigators should perform a more sophisticated analysis of macrophage subset activation status in the lungs of mice infected with influenza using WT and Acod1-/- mice. | While the connection between higher expression of ACOD1 in M2 macrophages is a very interesting initial observation, we can at this point not perform the required mouse infection experiments to investigate this question, as a new animal research application would be required (which would take several months to be approved by the regulatory authorities). In addition, we feel that the depth required to address this question sufficiently would go beyond the scope of the present paper. We therefore need to leave this as an interesting hypothesis-raising observation. We added text to the Discussion stating some implications of this interesting finding  Lines 572-576 |
| Furthermore, no analysis of viral titers is performed in Acod1-/- mice. Is the increased death and morbidity observed in Acod1-/- mice due to increase in viral replication? This should be directly demonstrated.  Analysis of HA transcripts in a cell line is not sufficient. | As explained above, we could not perform any additional in vivo experiments with the KO mice. Instead we tried to demonstrate presence of the virus by IHC for NP (using 2 different antibodies) in sections from the existing paraffin blocks from the exp. shown in Fig. 1. Unfortunately, the results were not specific enough to allow for a quantitative comparison. (also see above)  Instead, we performed a DI treatment experiment of IAV-infected WT mice, which is more relevant from the point of view of drug development. This experiment showed that the treatment greatly reduced lung inflammation but did not affect viral titers, whereas survival was significantly greater in the treated mice (Fig. 11). (also see above)  We respectfully disagree that we only presented HA transcripts in a cell line, as we used two different cell lines (nonproductive vs. productive infection), also showed HA mRNA in PBMC, and viral titers in primary lung tissue.  Nonetheless, we appreciated the reviewer’s concern and measured viral titers in an additional experiment using A549 cells. The results indeed greatly strengthen our paper, as the reduction of viral titers by itaconate and 4OI in A549 cells clearly shows that there is no increase in viral infectivity despite the reduction in IFN responses (Fig. 11). Additionally, the experiment gives first indications that 4OI interferes with viral infectivity at the post-RNA level in this cell type. |
| An analysis of the cytokines in the BAL and lungs using multiplex (luminex) approach would also be beneficial to understand the mechanisms that are important for the observed protection in Acod1 deficient mice. | We respectfully correct the reviewer in that *Acod1* deficient mice were not protected but were more susceptible. We do agree that it would be very interesting to measure inflammatory mediators in BAL or lung. However, we needed to prioritize additional experiments for this project, and future in vivo experiments will need to focus on the translational development of 4OI and related molecules. We added a statement to Discussion regarding a better understanding of the mechanisms of the increased susceptibility of the ACOD1 KO mice.  Lines 566-569. |
| The study suffers from repeated genomic studies performed on several different cell lines with similar results. Pretty much all that data can be moved to the supplementary section. The exact reasons for using so many different cells lines is not clear and does not add significance to the study. | In our opinion, using diverse cell types is a particular strength of our study, as they demonstrate that the compounds have general (cell-type independent) but also cell-type specific effects. These results will also help interpret results of future organismal studies.  In order to streamline the paper, we did move the GO term analyses of dTHP1 and A549 cells to the Supplement (new Fig. S6 and S12). |
| The paper is overtly unfocused and repetitious and hard to understand what the exact conclusions are. | We have tried to bring out the major conclusions better, for instance by adding more text about conclusions throughout Results and Discussion. In our opinion, the major conclusion is that compounds similar to 4OI would be the most promising candidates for further drug development, which is now clearly stated at the end of Discussion. |
| Figs 2 and 5 have several parts missing, thus, it is hard to evaluate this study when two main figures have several parts missing. | **Fig. 2:** due to an editing error the text still contained references to each individual panel in the figure, whereas only A and B were used in the figure. We corrected this in the text. We also added definitions of the virus strain abbreviations to the legend.  **Fig. 5:** same editing error as Fig. 2, which is now corrected. |
| The paper will benefit if the authors describe some of the studies better in the results section. It is difficult to assess exactly what was and done in each experiment. For example how were the M1 vs M2 macrophages generated? | We added a sentence to Results about how the M1 vs. M2 macrophages were generated (this was previously only described in Methods). Lines 169-171.  We also we tried to make the paper flow better by adding more text in each section of Results about the motivation underlying each set of experiments and making sure there is a brief conclusion at the end of each section. |
| FigS3- Please annotate the heatmaps for that entire cluster on the bottom. It has some important genes like TGFb and S100a8, the importance of these genes is not discussed. | We amended the figure accordingly.  We added a sentence to Results underscoring that the 2 compounds exert common and unique effects, including the unique downregulation of *TGFB* and *S100A8* mRNA (lines 202-204). We would rather not go into more detail about the effect on these genes in order to limit length of the manuscript. |
| FigS4- Can the conditions be put together. Heat maps throughout this paper have confusing color scheme, can they change it to make it black and white for one or the other? | We would rather not put the conditions together because the aim of using the unsupervised hierarchical clustering analysis was to reveal common and unique features of the treatments. However, we adopted a coloring scheme based on B&W with written treatment labels, which we also applied to all other heat maps in the paper. This figure is now much clearer, and we thank the reviewer for this suggestion. |
| Fig-4: This figure is too busy, needs to be simplified with a better form. Difficult to follow along | These stripcharts have become a popular way to visualize enrichment analyses. We therefore hesitate to change this format. However, for purpose of clarification we added two sentences in the Legend providing more background information. We also printed the most relevant GO terms in bold to make it easier for the reader to find them. We hope that the message is now clearer. This figure has been moved to the Supplement (S6). |
| **Part II – Major Issues: Key Experiments Required for Acceptance** | |
| **Reviewer 1** |  |
| Reviewer #1: 1. The authors stated that “chemical variants of itaconate, rather than its native form, will take the lead in further translational development to the bedside” (line 428). The “cytoprotective properties” and the mechanism of action of itaconate and chemical derivatives are not well understood. It became clear that itaconate and itaconate derivatives have very different impacts on metabolism (specifically SDH inhibition, see Artyomov lab PMID: 32694786 ). As such, the primary mechanism of action is not well understood. At times, the authors may reframe their statements; for instance, “both compounds likely exert cytoprotective effects by reducing ROS generation via inhibition of SDH.” (line 102). | We corrected the statement about SDH inhibition being a common cytoprotective mechanism, as the Artyomov paper (Swain, Bambouskova et al. 2020) clearly showed that DI and 4OI do not inhibit SDH (lines 102, 483-485). Incidentally, this also agrees with our own unpublished data in dTHP1 and A549 cells.  We added a table to the Discussion, which summarizes shared and unique features of the 3 compounds.  Table 1. |
| 2. Interestingly, D2 mice models have decreased ACOD1 expression compared to B6 mice. Does reduced ACOD1 expression led to reduced itaconate levels in the D2 animal model? Expression level does not reflect metabolite level, and itaconate concentrations might be similar in both animal models. If itaconate levels are identical, ACOD1/itaconate might not be the primary driver for the different disease severity described in the two animal models.  Further, some quantification of intracellular itaconate levels would be beneficial to better understand the treatment doses chosen for this study as 25 mM or 40 mM extracellular itaconate seems to be very high.  Are those concentrations physiological relevant? Specifically, which itaconate concentrations are achieved in macrophages and PBMCs upon infection (Fig. 2)? | We respectfully correct this reviewer in that the more susceptible DBA/2J mice have higher *Acod1* expression. However, the question whether this translates into higher Ita levels is still valid. We performed a new infection of B6 and D2 mice, which indeed showed that itaconate concentrations were higher in D2 mice on day 3 p.i. and correlated well with expression of Acod1 mRNA and viral HA mRNA.  Fig. S1. Lines 152-154  Nonetheless, we added a sentence to Discussion that increases in ACOD1 mRNA expression does not necessarily translate into corresponding differences in itaconate.  Lines 551-553  Meanwhile, we have measured itaconate concentrations in LPS/IFNg stimulated dTHP1 cells (500 μM). This concentration is clearly much lower than the itaconate concentrations added to the cells (20 or 40 mM), which are expected to result in intracellular concentrations of about 10-15 mM (our unpublished observations). However, we do not think this is a problem since the work on exogenously administered itaconate was not geared toward elucidating mechanisms of endogenous itaconate but to demonstrate proof-of-concept of pharmacological applications.  On the other hand, the concentration of itaconate resulting from transient transfection of ACOD1 into A549 cells is close to the above-mentioned 500 mcM and therefore is in the physiologically relevant range.  Lines 476-478 |
| 3. Itaconate derivatives seem to decrease ACOD1 expression levels (Fig 8). Some effects observed by treatments with itaconate derivatives might be due to decreased endogenous itaconate synthesis. On the other hand, 4OI might be converted into itaconate (Hooftman et al. 2020, study with 13C 4OI). How do itaconate levels change in response to DMI and 4OI treatments?  Further, the authors may want to repeat some key experiments in IRG1 KO cells treated with DMI or 4OI to decipher the impact of itaconate derivatives compared to endogenous itaconate levels. | To obtain correlations with all 3 compounds (i.e. including Ita), we treated IAV infected dTHP1 cells with Ita, DI and 4OI and measured ACOD1 mRNA. There is a negative correlation with the potency of the anti-inflammatory effect (4OI>DI>Ita) and ACOD1 expression (Ita>DI>4OI). Endogenous itaconate levels therefore most likely decrease with DI and 4OI administration. However, we do not think this is functionally relevant for the anti-inflammatory effect, but is more likely a manifestation of differences in reduction of IFN-I signaling by the compounds.    Lines 533-536, Fig. 6B  This hypothesis is supported by the results of the suggested experiment featuring DI treatment of WT vs. KO Acod1 cells (BMDM): CXCL10 reduction by DI was essentially the same in WT and KO cells. (lines 237-248, Fig. 4). This experiment was performed both with IAV infection and with IFNg stimulation, and the results were the same. |
| 4. The authors pretreated cells for 24 h before infection (Fig 3) for most of their studies. Are similar results achieved with acute treatments (as opposed to pretreatments)? Pretreatments might be clinically challenging. | In the DI treatment experiment of BMDM from WT and KO mice, we compared pretreatment only against pre- and acute treatment. The combination of pre+acute was more effective.We performed an experiment in dTHP1 cells comparing pretreatment only vs. acute treatment only, which showed that pretreatment alone had a strong effect with 4OI but none with Ita, whereas both showed CXCL10 reduction in acute treatment.  Lines 237-257, Fig. 4.  While these results further underscore the differences between the compounds, the clinical interpretation is complex. Regarding clinical relevance of pretreatment: even when treatment is first initiated in a putative patient with a viral infection, the patient’s cells likely experience a mixture of pre- and acute treatment. Many infected cells will take up the drug for the first time, but also a lot of cells will take up the drug before they are infected by the spreading virus. As the length of treatment increases and the drug has reached steady-state concentrations throughout the body, newly generated cells will likely first encounter the treatment and then the spreading virus. |
|  |  |
| **Reviewer #2:** |  |
| 1. At times, the GO/KEGG enrichment analyses provide more distraction than clarity. It is unclear why some GO terms were included in the main figures, while others were placed in the supplement.  For example, why include “excretion” in Fig. 4 while putting “Influenza A”, “RIG-I-like signaling”, and “TNF signaling” in Fig. S5?  As shown in Fig. S10 and S16, why would IAV infection show a greater enrichment for “Herpes simplex infection” and “Hepatitis C” than “Influenza A”? The authors may want to re-examine if including all of the GO/KEGG analyses is necessary. | We used both GO term and KEGG pathway enrichment analyses because the results complement each other. In a way they also provide some validation of each other as they are based on non-identical data sets and paradigms. GO terms provide a broader overview, whereas KEGG pathways are often more focused. Since all enrichment analyses (except those based on scRNAseq) are now in the Supplement, we would prefer to keep both. Now all GO term and KEGG pathway analyses are found in the supplement.  “Excretion” is in Fig. 4 (now S6) because it is a defined GO term. On the other hand “Influenza A”, “RIG-I” and “TNF” are among the defined KEGG pathways which are shown in Fig. S5 (now S12). As these are all responses to viral infections, there is considerable overlap in the regulated genes, and the genes which, according to KEGG, are part of the responses to HSV could also be assigned to “Influenza A”. This is a recognized weakness of current KEGG annotations. |
| 2. This is more of a comment than a major criticism. The authors expend a great deal of effort comparing and contrasting Ita treatment to DI or 4OI treatment. Since this work is descriptive work, it provides little insight into potential mechanistic differences between their modes of action. | We have performed additional experiments that provide a better understanding of the common and different immunomodulatory/anti-viral effects of the 3 compounds features and the underlying mechanisms.  pSTAT1 inhibition: Fig. 6A  pAKT inhibition: Fig. 6A  ROS reduction: Fig. 6C  DI vs. 4OI, effects on cytokine release from dTHP1 cells (Fig. S5B)  Reduction of progeny virions but not viral RNA (A549 cells): Fig. 11.  For instance, the fact that Ita upregulates but DI and 4OI downregulate pAKT may be one explanation for the divergent effects of Ita, pSTAT1 inhibition correlates with their ability to inhibit IFN-I responses (4OI>DI/Ita), whereas anti-ROS effects of the compounds are similar.  The differential effects of the compounds on viral titers (4OI>>Ita>DI) and lack of an effect on viral RNA in this cell type are among the most interesting additional data. Although there are plausible hypotheses for the underlying mechanism(s), it would (in our opinion) go beyond the scope of the current paper to test them experimentally at a sufficiently robust level.  We added a table to the manuscript contrasting major features of the 3 compounds and added text to Discussion highlighting the differences among the three compounds.  Table 1. |
| However, the authors’ data reveal interesting observations regarding the potential effects of DI and 4OI treatment on ACOD1 expression (and possibly intracellular Ita concentrations) that is largely ignored. Fig. 8 provides the best observation in this regard. In Fig. 8, the authors show that IAV infection induces the expression of ACOD1 in PBMCs (a finding that confirms observations in Fig. 2). Ita treatment further promotes ACOD1 expression in infected cells. However, DI and 4OI reduce ACOD1 expression in infected cells and uninfected cells. A similar observation is found in Fig. 9, where infection increases ACOD1 expression in monocytes, but DI treatment reduces it. Did the authors measure intracellular Ita concentrations in these conditions to see if they correlate with ACOD1 expression? It would be interesting to know if the authors think that DI and 4OI effect on ACOD1 expression (and presumably intracellular Ita concentrations) is a possible mechanism that explains the differences they observed between Ita treatment and DI/4OI treatment. | We thank the reviewer for these interesting thoughts. In order to check whether upregulation of ACOD1 is a common feature of Ita treatment, we performed a dedicated experiment in which we treated IAV-infected dTHP1 cells with the three compounds. All three reduced ACOD1 expression, and the extent of reduction (4OI>DI>Ita) roughly correlated with their ability to inhibit IFN signaling (4OI>DI/Ita).  Fig. 6B  To check whether endogenous Ita synthesis is required for an Ita treatment response, we applied DI to IAV infected or IFNg stimulated BMDMs from WT and Acod1 KO mice. The treatment response was nearly identical independent of the genotype, indicating that at least in this viral scenario endogenous Ita is not a notable determinant of the treatment response.  Fig. 4  Based on these two new observations, the simplest interpretation of the raised (or at least unchanged) ACOD1 expression in Ita-treated PBMC is that it is not causal to the different treatment effects of Ita in PBMC. A more plausible explanation is that it is an indicator of a paradoxical stress response of the cells to exposure to exogenously administered Ita. Indeed, we showed some time ago (Preusse et al. BMC Microbiology 2013) that the stress from mock infection and anesthesia can induce ACOD1 expression in mouse lung. Therefore, we consider induction of ACOD1 by Ita a biomarker for, but not a cause of, an incomplete treatment response in the PBMC. We added text about this to Discussion.  Lines 526-536 |
|  |  |
| **Reviewer #3** |  |
| The authors should attempt to at the very least perform these studies in vivo in the mouse model and use flow cytometry to determine how the inflammatory response is affected by these treatments and if downregulation of CXCL10 and CCL2 transcripts matches actual reduction in protein production and a concomitant decrease in myeloid cell (monocytes, neutrophils etc) recruitment. | We examined the effect of DI on survival, viral titers and lung inflammation in a new in vivo experiment (Fig. 11). The immunological consequence of DI-dependent decrease in CCL2 levels has already been published by Wang et al, who used a colitis-tumor model and found that DI reduced IL-1b and CCL2 and reduced recruitment of macrophages (Wang, Li et al. 2020). We added this finding to the text.  Lines 565-566  Please see our responses above for more details and the scientific and ethical justification for not going ahead with additional in vivo experiments about immunological mechanisms of DI. |
| There are many subsets of macrophages in the lungs that mediate distinct functions, the investigators should perform a more sophisticated analysis of macrophage subset activation status in the lungs of mice infected with influenza using WT and Acod1-/- mice. | We agree with the reviewer that this would be a very interesting point to examine, and we added it to the Discussion. However, even though the data would clearly enrich the paper, we do not think that they are critical for its overall message, as the paper does not have the role of endogenous itaconate as the main focus.  Lines 572-576 |
| Furthermore, no analysis of viral titers is performed in Acod1-/- mice. Is the increased death and morbidity observed in Acod1-/- mice due to increase in viral replication? This should be directly demonstrated. Analysis of HA transcripts in a cell line is not sufficient. | An indirect indication that it is not due to increased viral replication lies in the new DI treatment studies, where survival was better in the treated mice even though viral titers did not change.  Even the original version of the manuscript contained data on HA transcripts in two cell lines and PBMC, and on viral titers in human lung. We now added the inhibitory effect on release of viral progeny from A549 cells. Taken together, the results show that viral infectivity does not increase despite the remarkable reduction in IFN responses. |
| An analysis of the cytokines in the BAL and lungs using multiplex (luminex) approach would also be beneficial to understand the mechanisms that are important for the observed protection in Acod1 deficient mice. | We respectfully correct the reviewer in that KO mice are not protected, but they are more susceptible.  We agree that the suggested experiments would enrich the paper further, but (as stated above) they are not critical for the overall message of the paper. We added text regarding this to Discussion.  Line 566-568 |
|  |  |
| **Part III – Minor Issues: Editorial and Data Presentation Modifications** | |
| **Reviewer #1:** |  |
| The authors may cite and discuss some key studies on itaconate derivatives, specifically work by Artyomov Lab (PMID: 32694786, impact on inflammation), Hooftman et a (PMID: 32791101, Cell Metab, inhibition of NLRP2 inflammasome activation by 4OI), and Sethy et al. (2019, J.Med.Chem, identification of itaconate derivates as potential anti-influenza agents). For instance, the current manuscript demonstrated that CXCL10 expression was decreased upon itaconate/derivative treatment, while work by Artyomov lab reported increased levels upon itaconate treatment in IRG1 KO conditions. | We have added these references to the paper and discuss our findings in their light (various passages).  In our experience, the effect of the compounds reverts from decreasing CXCL10 to increasing CXCL10 as we increase the dose and approach levels that are not overtly toxic but exert cell stress (unpublished). The increase seen by Artyomov lab may thus reflect a stress response. Indeed, IFN-I responses can be induced by cell stress (Preusse, Tantawy et al. 2013). This agrees with the study by (Muri, Wolleb et al. 2020) (Discussion, line 457), which argues that immunomodulatory effects of Nrf2 activators can be dose-dependent. Artyomov used BMDM from Irg1 KO mice. It is possible that these cells are susceptible to developing cell stress even at the low concentration of Ita used. This point is addressed in new text in Discussion. |
| **Reviewer #2:** |  |
| 1. The manuscript would benefit from a model or schematic to summarize the most salient data clarify their overall conclusion(s). | We prepared a table summarizing shared and distinct features of the 3 compounds.  Table 1 |
| 2. In Fig. 7A, Ita and DI treatments were found to have no effect on virus titers in the human lung tissue explant model. This observation is mostly ignored in the rest of the manuscript. This is a significant finding; the authors should include this information in the Abstract and Discussion. | We expanded the section on anti-viral effects in Discussion  Lines 488-504 |
| 3. The authors discuss the cytotoxicity of Ita, DI, and OA4 treatment on several cell types on several occasions, but no data were presented. The authors need to include the data from the cytotoxicity assays in the manuscript. | We added cytotoxicity data (MTT assay) for the three compounds on THP1 and A549 cells. They demonstrate that the concentrations used by us were always in the non-toxic range. An additional finding of interest is that the differences in toxicity of the compounds also differ according to the cell type.  Figure S2 |
| 4. At the end of the description for Fig. 8 the authors state that “the miscellaneous effects of 4OI on gene expression in this model, we subsequently focused on DI to decipher effects at the single-cell level.” However, 4OI had what appears to be a stronger anti-inflammatory property than DI, while having the added benefit of reducing HA expression. Could the author expand on what exactly they mean by “miscellaneous effects of 4OI on gene expression” that caused them to focus on DI instead of 4OI? | We rephrased this part of Results to reflect more accurately that this experiment would reveal effects of an itaconate derivative on cell gene expression in the absence of a notable antiviral effect.  Lines 367-369 |
| 6. The authors should include a discussion/reference to Sethy, et al., “Design, Synthesis, and Biological Evaluation of Itaconic Acid Derivatives as Potential Anti-Influenza Agents” <https://doi.org/10.1021/acs.jmedchem.8b01683> | We discuss this report now in the context of the reduction of IAV titers by 4OI and Ita.  Lines 493-496 |
| 7. Could the authors clarify why they used only female mice? | We chose to study only one sex in order to exclude sex as a confounding variable. Female mice were used in most studies on host genetic background performed at our institution. In addition, animal caretakers requested to use female mice because of easier handling. |
| 8. Lines 242-244 “infection with IAV led to brisk transcription of viral HA and host CXCL10 mRNA in a 72 h time course (Fig. S11), whereas ACOD1 mRNA levels did not change significantly.” No ACOD1 data is presented in Fig. S11, and this statement appears to be in contradiction to data shown in Fig. S12. | Increased ACOD1 expression resulted only in a subset of tissue pieces; statistical significance was therefore not reached. The reason is most likely that ACOD1-expressing myeloid cells may be rare in some regions of these terminally diseased lungs.  Fig. S11 (old) refers to lung tissue from patients with terminal lung failure, requiring transplantation, whereas S12 (old) is the re-analysis of a dataset derived from healthy lung tissue. |
| 9. The figure legend for Fig. 1G is missing details. Are the images shown from WT or KO mice? Also, there is no reference to “Fig. 1G” in the text of the result section | Corrected |
| 10. Throughout the manuscript “FC” is used in the figures. But it is often unclear exactly how the fold change was calculated. A short description in the figure legend each time “FC” appears would help | We added the definition of FC to all figures as indicated. |
| 11. The figure legend for Fig. 3 is missing a description of the concentrations of DI used in the experiment. | The reviewer probably overlooked the fact that concentrations of DI were stated in the legend for all three experiments. |
| 12. A description of the generation of the A549 cells expressing ACOD1 appears to be missing from the Materials and Methods section. | We added this to Methods  Lines 607-611 |
| 13. Fig. 2 only has a panel A and B. However, the text describing Fig. 2 references Figure C-E. This makes the description on lines 151-163 somewhat unclear. | We deleted the references to C-E. |
| 14. The authors need to describe Fig. 3A in the text of the Results section. | Done |
| 15. Line 212: “IA” do they mean itaconate? In other places, they abbreviate it “Ita”. | Done |
| 16. Figure 5 only has a panel A and B. However, the text describing Figure 5 references Figure C-H. This makes the description on lines 206-223 somewhat unclear. | Corrected |
| 17. It would help if additional details regarding the infection were included in the figure legends that included M.O.I. and time of infection, particularly when performing reanalysis of published data (for example, Fig. 1A). | We added information about infectious doses and time post infection to all legends as indicated. |
| 18. Line 341. “ell” typo | Corrected |
| 19. Line 763: “Go” typo | Corrected |
| 20. Reference 50 needs greater detail | We replaced it with an up-to-date reference to a peer-reviewed article. |
|  |  |
| **Reviewer #3:** |  |
| The paper will benefit if the authors describe some of the studies better in the results section. It is difficult to assess exactly what was done in each experiment. For example how were the M1 vs M2 macrophages generated? | In general, we tried to add more background information at the beginning of each section in Results to clarify the motivation for each experiment.  We added more information about generation of the M1 and M2 macrophages (Lines 169-171). |
| FigS3- Please annotate the heatmaps for that entire cluster on the bottom. It has some important genes like TGFb and S100a8, the importance of these genes is not discussed. | We labeled the figure accordingly. And also briefly mentioned TGFb and S100A8 in the text. We decided to not add further discussion of this interesting finding, though, in order to limit the amount of new text in this revision.  Lines 207-209 |
| FigS4- Can the conditions be put together. heat maps throughout this paper have confusing color scheme, can they change it to make it black and white for one or the other? | We changed the color scheme to a B+W scheme, writing the treatments into the label boxes. We adopted this scheme for all other heat maps in the paper in order to improve recognition value.  We did not put the conditions together in order to preserve the results from hierarchical clustering about relationships among the samples |
| Fig-4: This figure is too busy, needs to be simplified with a better form. Difficult to follow along | We added the + and – legend also to the top of the figure and printed the most relevant GO terms (i.e. related to inflammation, up in IAV infection, down by Ita and DI) in bold. |
|  |  |
| PLOS authors have the option to publish the peer review history of their article ([what does this mean?](https://journals.plos.org/plospathogens/s/editorial-and-peer-review-process#loc-peer-review-history)). If published, this will include your full peer review and any attached files.  If you choose “no”, your identity will remain anonymous but your review may still be made public.   **Do you want your identity to be public for this peer review?** For information about this choice, including consent withdrawal, please see our [Privacy Policy](https://www.plos.org/privacy-policy).  Reviewer #1: No  Reviewer #2: No  Reviewer #3: No  Figure Files:  While revising your submission, please upload your figure files to the Preflight Analysis and Conversion Engine (PACE) digital diagnostic tool, [https://pacev2.apexcovantage.com](https://pacev2.apexcovantage.com/). PACE helps ensure that figures meet PLOS requirements. To use PACE, you must first register as a user. Then, login and navigate to the UPLOAD tab, where you will find detailed instructions on how to use the tool. If you encounter any issues or have any questions when using PACE, please email us at [figures@plos.org](mailto:figures@plos.org).  Data Requirements:  Please note that, as a condition of publication, PLOS' data policy requires that you make available all data used to draw the conclusions outlined in your manuscript. Data must be deposited in an appropriate repository, included within the body of the manuscript, or uploaded as supporting information. This includes all numerical values that were used to generate graphs, histograms etc.. For an example see here on PLOS Biology: <http://www.plosbiology.org/article/info%3Adoi%2F10.1371%2Fjournal.pbio.1001908#s5>.  Reproducibility:  To enhance the reproducibility of your results, we recommend that you deposit your laboratory protocols in protocols.io, where a protocol can be assigned its own identifier (DOI) such that it can be cited independently in the future. Additionally, PLOS ONE offers an option to publish peer-reviewed clinical study protocols. Read more information on sharing protocols at <https://plos.org/protocols?utm_medium=editorial-email&utm_source=authorletters&utm_campaign=protocols>    *In compliance with data protection regulations, you may request that we remove your personal registration details at any time.* [*(Remove my information/details)*](https://www.editorialmanager.com/ppathogens/login.asp?a=r)*. Please contact the publication office if you have any questions.*  <Pessler | |

**References**

Bambouskova, M., L. Potuckova, T. Paulenda, M. Kerndl, D. A. Mogilenko, K. Lizotte, A. Swain, S. Hayes, R. D. Sheldon, H. Kim, U. Kapadnis, A. E. Ellis, C. Isaguirre, S. Burdess, A. Laha, G. K. Amarasinghe, V. Chubukov, T. P. Roddy, M. S. Diamond, R. G. Jones, D. M. Simons and M. N. Artyomov (2021). "Itaconate confers tolerance to late NLRP3 inflammasome activation." Cell Rep **34**(10): 108756.

Muri, J., H. Wolleb, P. Broz, E. M. Carreira and M. Kopf (2020). "Electrophilic Nrf2 activators and itaconate inhibit inflammation at low dose and promote IL-1beta production and inflammatory apoptosis at high dose." Redox Biol **36**: 101647.

Preusse, M., M. A. Tantawy, F. Klawonn, K. Schughart and F. Pessler (2013). "Infection- and procedure-dependent effects on pulmonary gene expression in the early phase of influenza A virus infection in mice." BMC Microbiol **13**: 293.

Swain, A., M. Bambouskova, H. Kim, P. S. Andhey, D. Duncan, K. Auclair, V. Chubukov, D. M. Simons, T. P. Roddy, K. M. Stewart and M. N. Artyomov (2020). "Comparative evaluation of itaconate and its derivatives reveals divergent inflammasome and type I interferon regulation in macrophages." Nat Metab **2**(7): 594-602.

Wang, Q., X. L. Li, Y. Mei, J. C. Ye, W. Fan, G. H. Cheng, M. S. Zeng and G. K. Feng (2020). "The anti-inflammatory drug dimethyl itaconate protects against colitis-associated colorectal cancer." J Mol Med (Berl) **98**(10): 1457-1466.
